# Supplementary material for: Piwi reduction in the aged niche eliminates germline stem cells via Toll-GSK3 signaling
Source: Nat Commun. 2020 Jun 19;11:3147. doi: 10.1038/s41467-020-16858-6 (PMC7305233; doi:10.1038/s41467-020-16858-6)
Supplement: Supplementary file 1 — Supplementary Information [file 41467_2020_16858_MOESM1_ESM.pdf]

Supplementary Information

Piwi Reduction in the Aged Niche Eliminates Germline Stem Cells via Toll-GSK3 Signaling

By Lin *et al.*

Supplementary fig. 1-20
Supplementary table 1-4

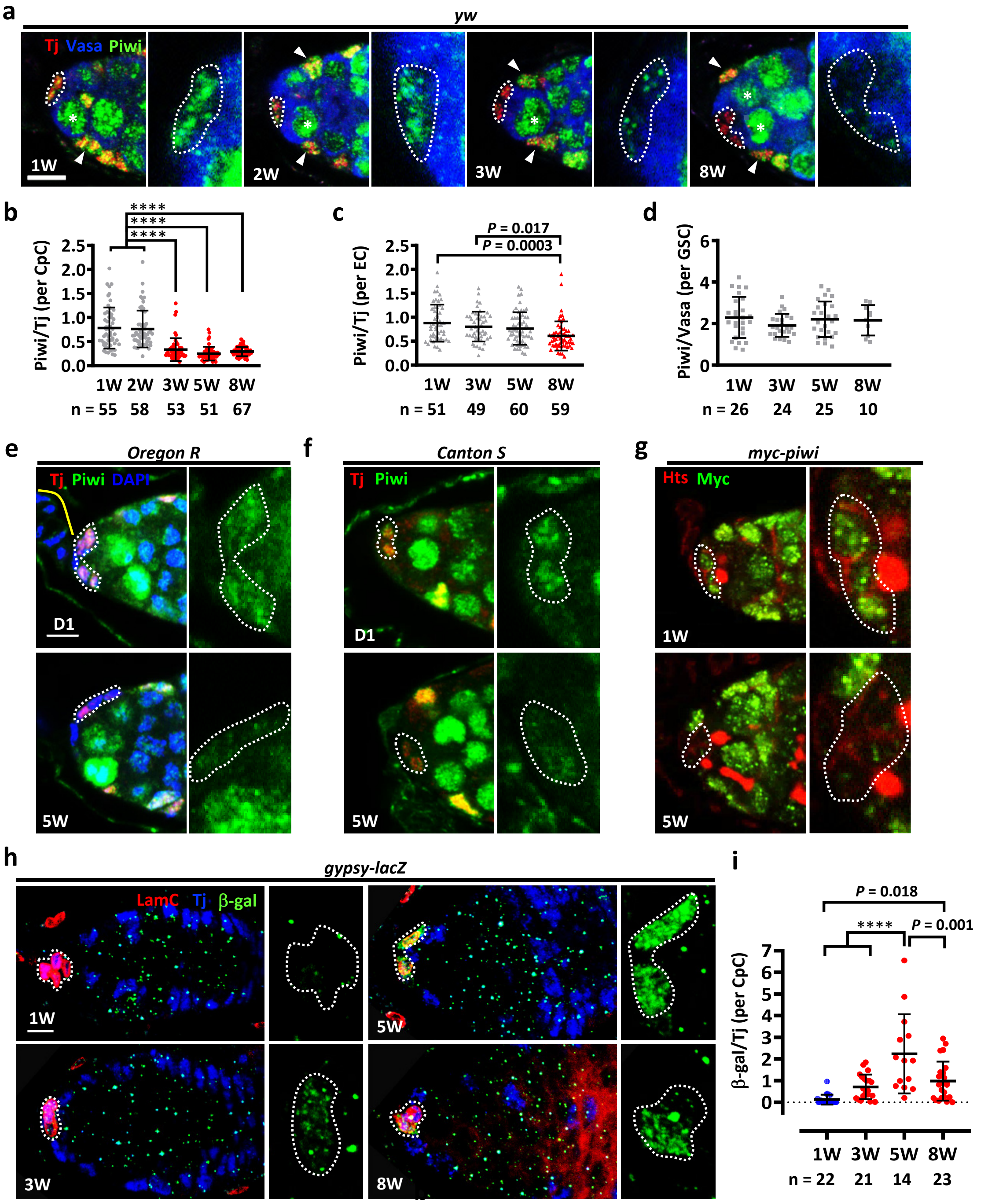

Supplementary figure 1. Piwi is decreased and *gypsy-lacZ* expression is elevated in the aged CpCs.

Supplementary Figure 1. Piwi is decreased and *gypsy-lacZ* expression is elevated in aged CpCs. a-d, Piwi expression is decreased in 3-week (W)-old cap cells (CpCs, dashed circles), slightly reduced in 8-week-old escort cells (ECs, arrowheads), but not changed in germline stem cells (GSCs, asterisks) of *yw* flies (green, Piwi; red, Tj for CpC and escort cell (EC) nuclei; blue, Vasa for germ cells). Relative expression of Piwi was normalized to Tj in CpCs (b) and ECs (c), or compared to Vasa in GSCs (d) at indicated ages. **e and f**, Piwi expression is reduced in 5-week-old cap cells of two wild-type *Drosophila* strains, *Oregan R* and *Canton S* (green, Piwi; red, Tj; blue, DAPI). Yellow line, TF cells. **g**, Myc-Piwi expression is reduced in 5-week-old CpCs of transgenic *myc-piwi* flies (green, Myc-Piwi; red, Hts for fusomes). **h**, The expression of *gypsy-LacZ*, the transposon reporter, is increased in CpCs (dashed circles) with age (green, b-gal for LacZ; red, LamC for CpC nuclear envelopes; blue, Tj). **i**, *gypsy-lacZ* is increased in aged CpCs. Relative expression of *gypsy-lacZ* was normalized to Tj in CpCs at indicated ages. Note that *gypsy-LacZ* expression is reduced in 8-week-old cap cells as compared to 5-week-old cap cells. This reduction might be due to the effects of aging on efficiency of *gypsy-lacZ* transcription or the responses of individual cap cells to aging might differ. Analyzed CpC (b and i), EC (c) and GSC (d) numbers (n) from biologically independent samples are showed below each graph**.** Data are presented as mean values +/- S.D.. One-way ANOVA was used for statistical analysis (b, c, d, i). ****P < 0.0001. Scale bar, 5 μm

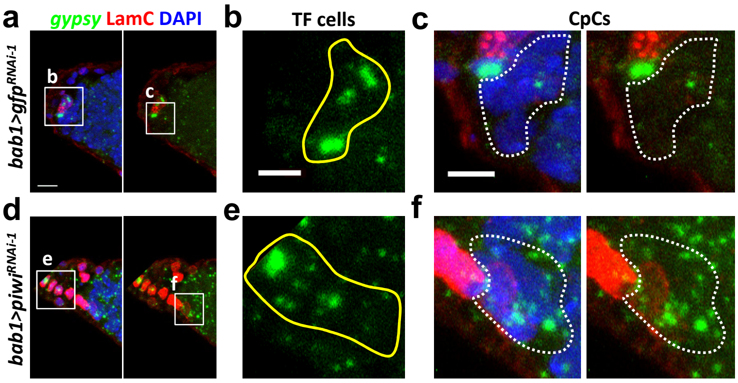

Supplementary figure 2. *gypsy* transcripts are up-regulated in Piwi-deficient CpCs. a and d, Two-week-old *bab1>gfp*$^{RNAi\text{-}1}$ (a) and *bab1>piwi*$^{RNAi\text{-}1}$ germaria (b) with *gypsy* transcripts (green), LamC (red, terminal filament (TF) and cap cell (CpC) nuclear envelopes) and DAPI (blue, DNA). b, c, e, and f are enlarged view of squares shown in a and d. Yellow lines outline TF cells, and white dashed lines outline cap cells. Scale bar in a and d is 5 µm, and in b and c are 2.5 µm. b and e are same magnification and share scale bar with b. c and f are same magnification and share scale bar with c.

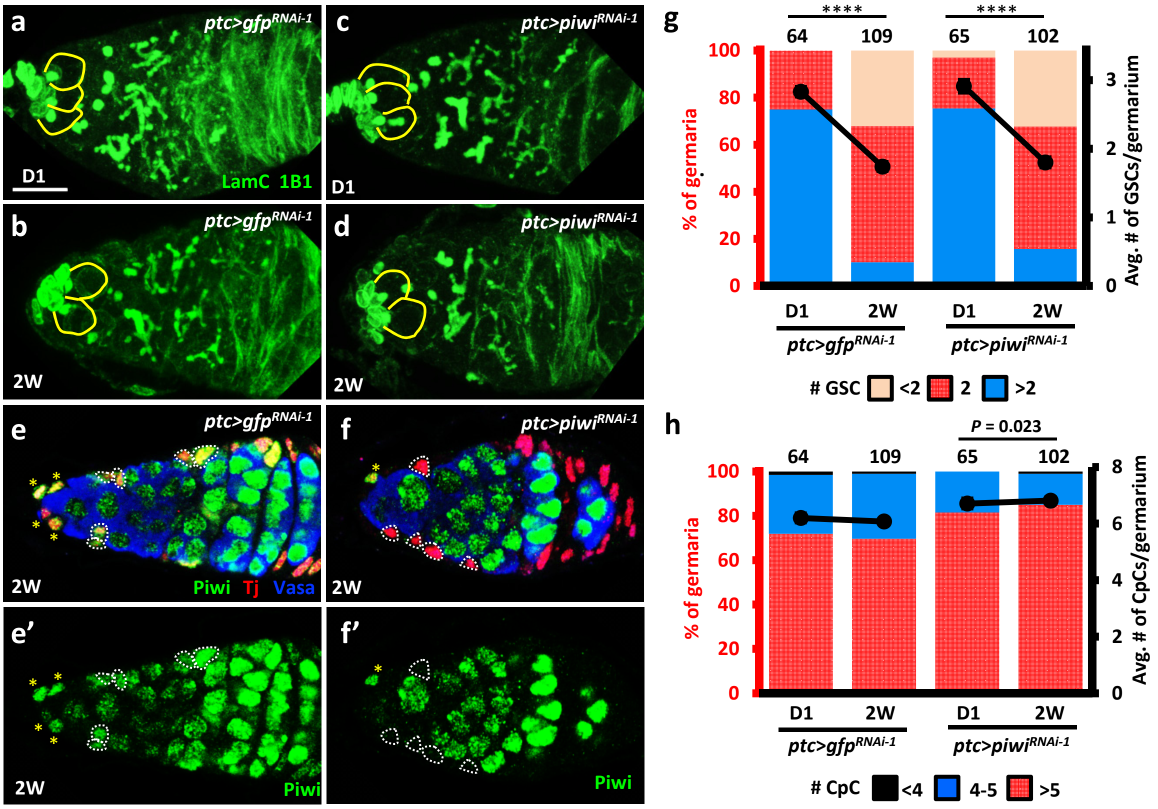

Supplementary Figure 3. Piwi is dispensable in adult escort cells for GSC maintenance. a-d, GSCs are maintained in the germarium carrying *piwi*$^{RNAi-1}$-expressing escort cells (ECs) driven by *ptc-GAL4* (an EC driver) compared to control (green, LamC for CpC nuclear envelopes and Hts for fusomes). GSC, yellow line. **e and f,** Expression of *piwi*$^{RNAi-1}$ in ECs efficiently decreases Piwi expression in 2-week (W)-old ECs (dashed circles) of *ptc>piwi*$^{RNAi-1}$ germaria, but not in CpCs (asterisks), as compared to the *ptc>gfp*^{RNAi} control germarium (green, Piwi; red, Tj for CpC and EC nuclei; blue, Vasa for germ cells). e' and f' show the Piwi channel only. **g**, GSC number is not affected in *ptc>piwi*$^{RNAi-1}$germarium. GSC number in day 1 and 2-week-old germaria of indicated genotypes. Percentage (%) of germaria (left Y-axis) indicates the proportion of germaria carrying 0, 1, 2, 3 or 4 GSCs. **h**, CpC number is slightly increased, but not reduced, after knocking down *piwi* in ECs for 2 weeks. CpC number in day 1 and 2-week-old germaria of indicated genotypes. Percentage (%) of germaria (left Y-axis) indicates the proportion of germaria carrying < 4, 4 or 5, and > 5 CpCs. The numbers of analyzed germaria are shown above each bar. Student's *t*-test was used for statistical analysis. Data are presented as mean values +/- S.E.M. (g and h). ****$P < 0.0001$. Scale bar, 5 μm.

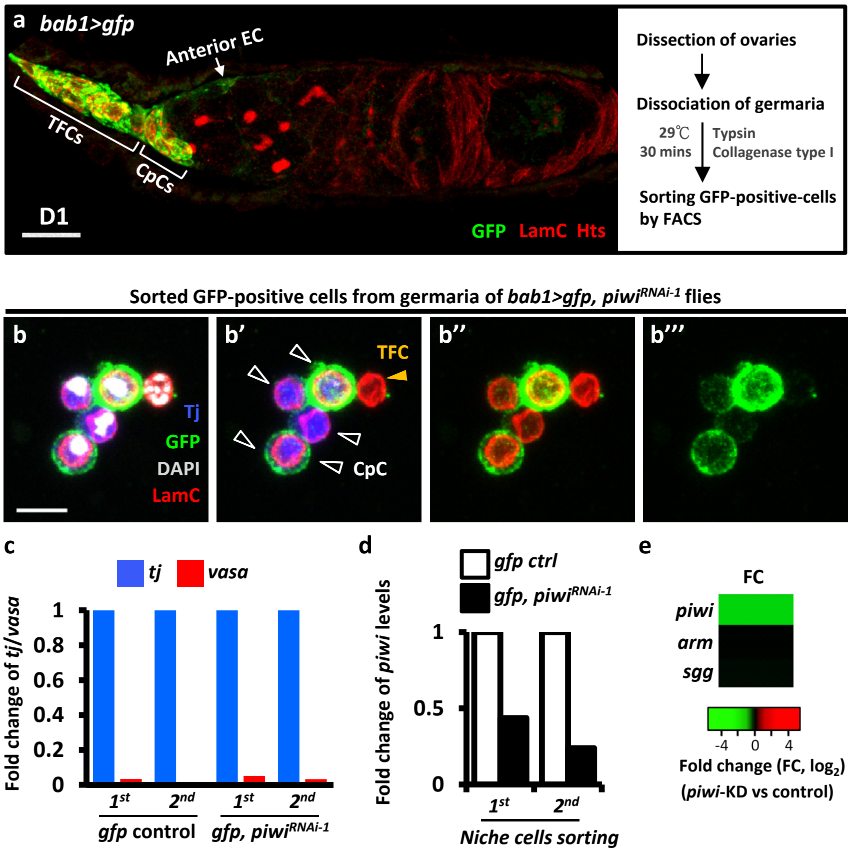

Supplementary Figure 4. Isolation of niche cells for transposon and transcriptome analysis. a, *bab1>gfp* and *bab1>gfp&piwi*$^{RNAi-1}$ germaria were dissociated by protease reaction and GFP-positive cells [mainly TF cells (TFCs) and CpCs] were sorted by FACS. Biological duplicates were used for analysis. Scale bar, 10 μm. **b-b''',** Sorted cells were stained with Tj (CpC and EC marker), LamC (TFC and CpC marker), GFP and DAPI (DNA). Most sorted GFP-positive cells were CpCs (LamC-positive and Tj-positive). Note that some TF cells (LamC-postive only) were also sorted. Scale bar, 5 mm. **c,** qPCR analysis showed that sorted niche cells had little, if any, germline contamination, indicated by the ratio of *tj* (soma marker) to *vasa* (germline marker). **d,** qPCR analysis showed *piwi* expression is reduced in sorted niche cells of *bab1>gfp&piwi*$^{RNAi-1}$ germaria compared to *bab1>gfp* control germaria. **e,** Transcriptome analysis of *piwi*-KD niche cells showed that *piwi* expression is reduced, but expression of *arm* or *sgg* are not, compared to *gfp* control.

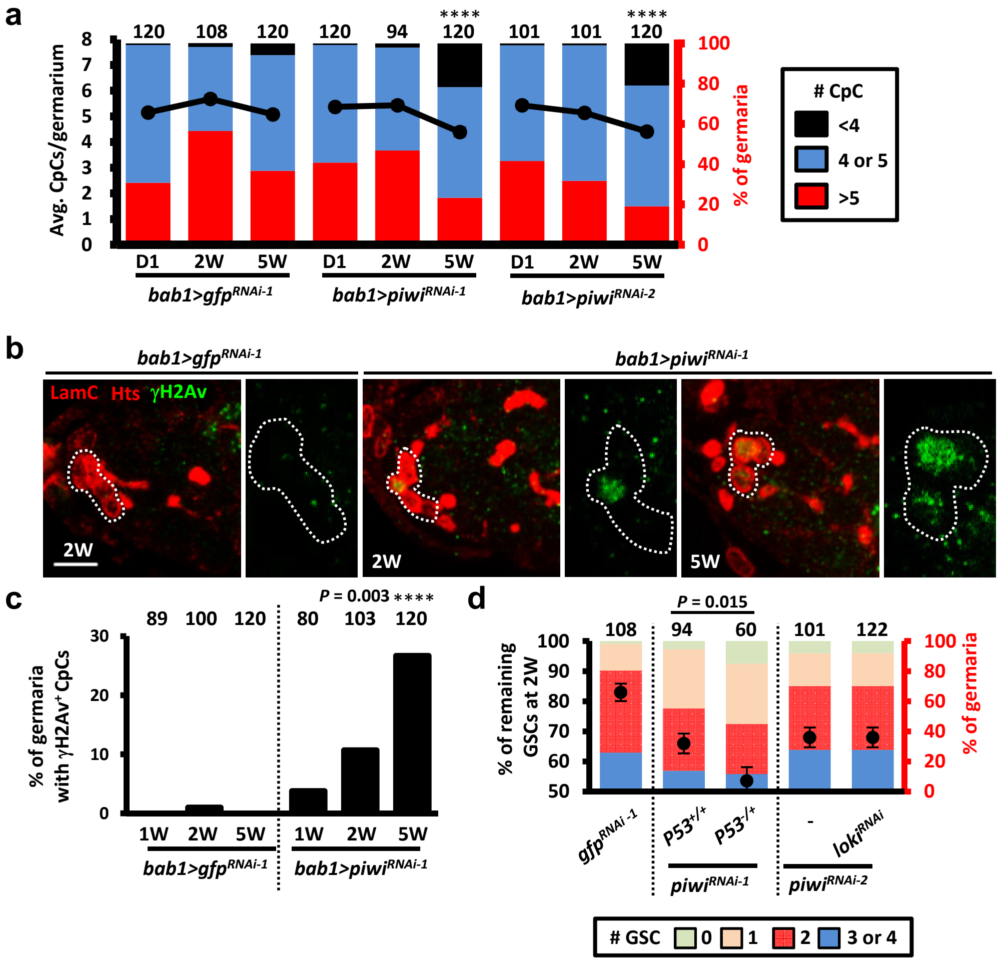

Supplementary Figure 5. DNA damage signaling does not contribute to GSC loss in germaria carrying Piwi-depleted CpCs. a, CpC numbers are slightly reduced in *piwi*-KD germeria of 5-week-old flies. CpC number in *bab1>gfp*$^{RNAi-1}$, *bab1>piwi*$^{RNAi-1}$, and *bab1>piwi*$^{RNAi-2}$ females at day 1 (D1), 2 weeks (2W), and 5 weeks (5W) after eclosion. **b,** gH2Av signals are increased in 2-week and 5-week (W)-old *bab1>piwi*$^{RNAi-1}$ niche with age (green, gH2Av; red, LamC for CpCs and Hts for fusomes). **c,** Percentage (%) of germaria containing gH2Av-positive (+) CpCs of *bab1>gfp*$^{RNAi-1}$ and *bab1>piwi*$^{RNAi-1}$ females at 1, 2, and 5 weeks after eclosion. Dashed circle, CpCs. Scale bar, 5 µm. **d,** Downregulation of DNA damage signaling pathway by either knocking down *loki* or introducing one copy of mutant p53 in the *piwi*-KD niche does not rescue GSC number. In fact, mutating one copy of p53 exacerbates GSC loss, indicating a requirement for p53 signaling in *piwi*-KD niche for GSC maintenance. GSC number in *bab1>gfp*$^{RNAi-1}$, *bab1>piwi*^{RNAi}, *bab1>piwi*$^{RNAi-1}$*;p53*$^{-/+}$, *bab1>piwi*$^{RNAi-2}$ and *bab1>piwi*$^{RNAi-2}$*;loki*^{RNAi} flies at 2 weeks after eclosion. Left Y axis, percentage (%) of remaining GSCs at 2 weeks normalized to GSC number at Day 1. Right Y axis, % of germaria carrying 0, 1, 2, 3 or 4 GSCs. Note that GSC number of *bab1>gfp*$^{RNAi-1}$, *bab1>piwi*$^{RNAi-1}$ and *bab1>piwi*$^{RNAi-2}$ are the same data as shown in fig. 1d. The numbers of analyzed germaria (a,c, d) are shown above each bar. Data are presented as mean values +/- S.E.M. (a and d). Student's *t*-test (a and d) and Chi-squared analysis (c) were used for statistical comparisons. ****$P < 0.0001$.

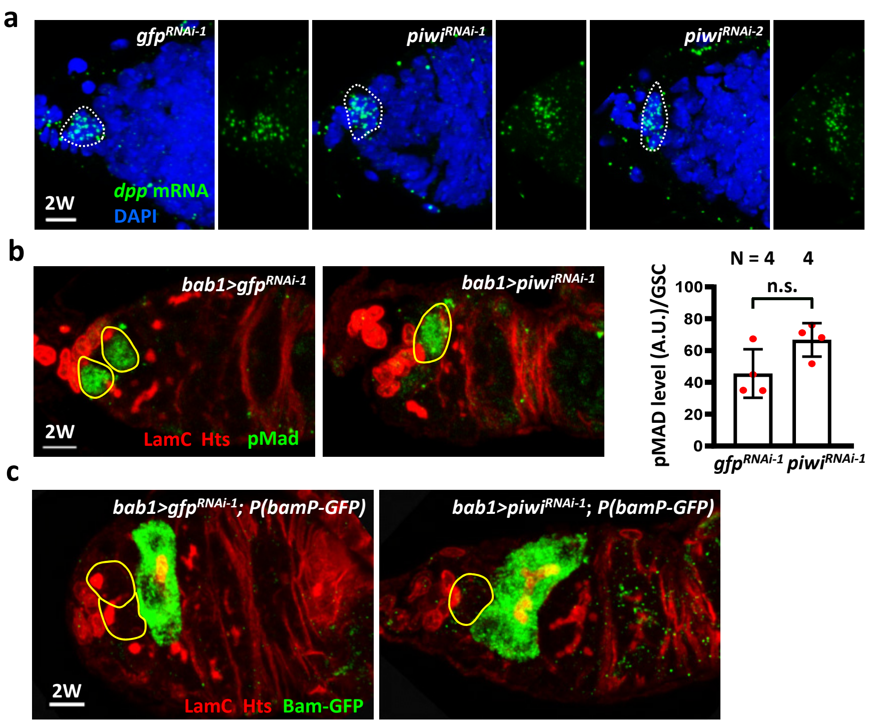

Supplementary Figure 6. Niche Piwi does not regulate Dpp production in the niche or Dpp signaling in GSCs. **a**, *dpp* mRNA expression is similar to controls in cap cells (dashed circle) of 2-week (W)-old *bab1>piwi^{RNAi-1}* and *bab1>piwi^{RNAi-2}* germaria (green, *dpp* mRNA; blue, DAPI). **b**, pMad expression as shown by mean of pMAD intensity from four independent experiments (N=4) in which 187 GSCs of control group and 130 GSCs of *piwi*-KD group are analyzed is not decreased in GSCs (yellow circles) of 2-week-old *bab1>piwi^{RNAi-1}* germaria (green, pMad; red, LamC for TF and CpC nuclear envelopes, and Hts for fusomes). **c**, Bam, a differentiation factor expressed in differentiated germ cells, (green, Bam-GFP; red, LamC and Hts) is not expressed in GSCs (yellow circles) of 2-week (W)-old *bab1>piwi^{RNAi-1}* germaria. P(*bamP-GFP)*, a transcriptional reporter of *bam* gene. The numbers of analyzed GSCs are shown above each bar (b). Student's *t*-test was used for statistical comparisons. Data are presented as mean values +/- S.D.. n.s., non-significant. Scale bar, 5 μm. A.U., arbitrary units.

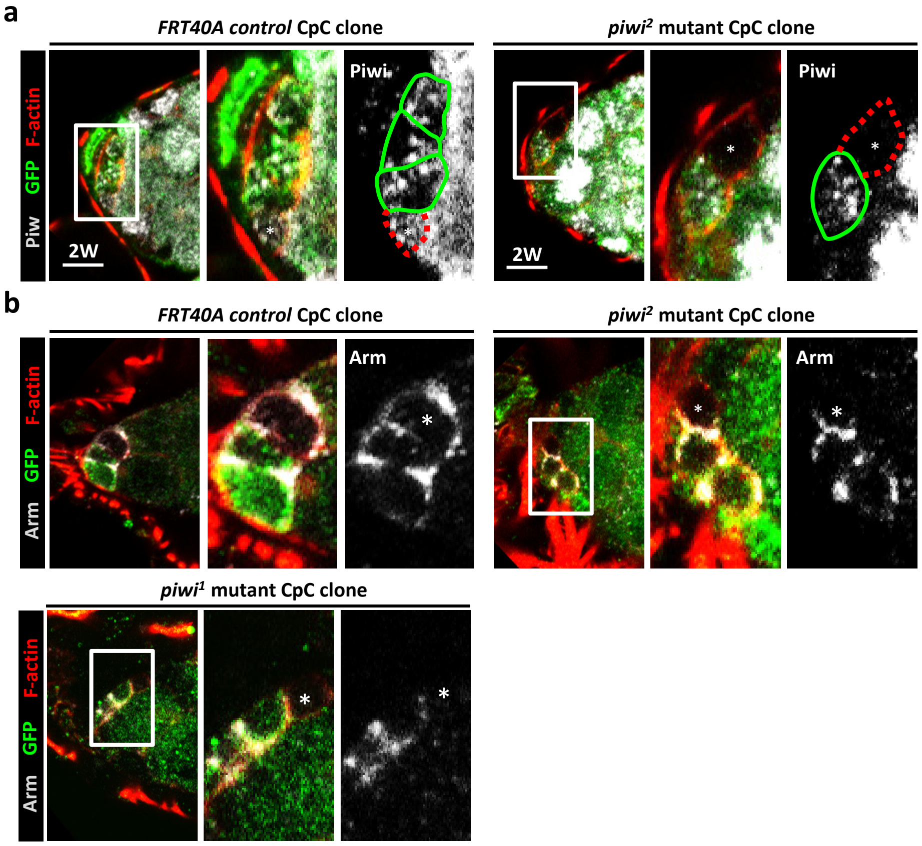

Supplementary Figure 7. Arm expression is reduced in *piwi* mutant CpCs. a, Piwi expression (white; red, F-actin; green, GFP) is reduced in *piwi*^{2} mutant CpC clone (right) compared to FRT control CpC clone (left). **b,** Arm expression (white; red, F-actin; green, GFP) is decreased in *piwi*^{2} mutant CpC clone (right) and *piwi*^{1} mutant CpC clone (bottom) compared to FRT control CpC clone (left). Green circle, GPF-positive clone. Red dashed circle and asterisks, GFP-negative clone. Scale bar, 5 µm.

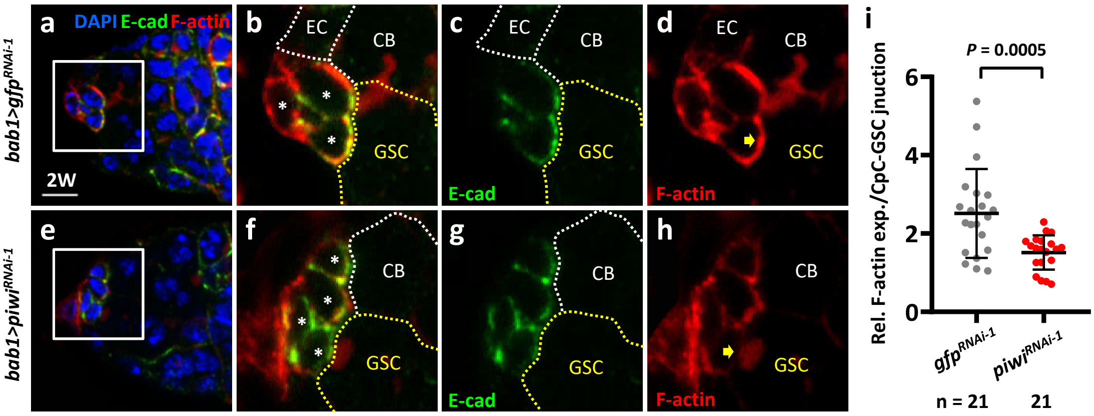

Supplementary Figure 8. Depletion of Piwi in the niche reduces F-actin expression at the niche-GSC junction. a and d, Two-week (W)-old *bab1>gfp*$^{RNAi\text{-}1}$ (a) and *bab1>piwi*$^{RNAi\text{-}1}$ germaria (e) stained for F-actin (red), E-cadherin (E-cad, green) and DAPI (blue, DNA). b-d and f-h are enlarged views of the boxed regions in a and e, showing merged (b and f), E-cad (c and g) and F-actin channels (d and h). F-actin signals are reduced at the junctions between CpCs and GSCs in the *bab1>piwi*$^{RNAi\text{-}1}$ germarium. Asterisks mark CpCs; yellow dashed circles outline GSCs; white dashed circles outline cystoblasts or escort cells; yellow arrows indicate CpC-GSC junctions. **i**, F-actin expression in the niche-GSC junction of indicated genotypes. Error bars, S.D. The numbers of analyzed CpC-GSC junctions are shown below the graph. Student's *t*-test was used for statistical comparisons. Data are presented as mean values +/- S.D. Scale bar, 5 μm.

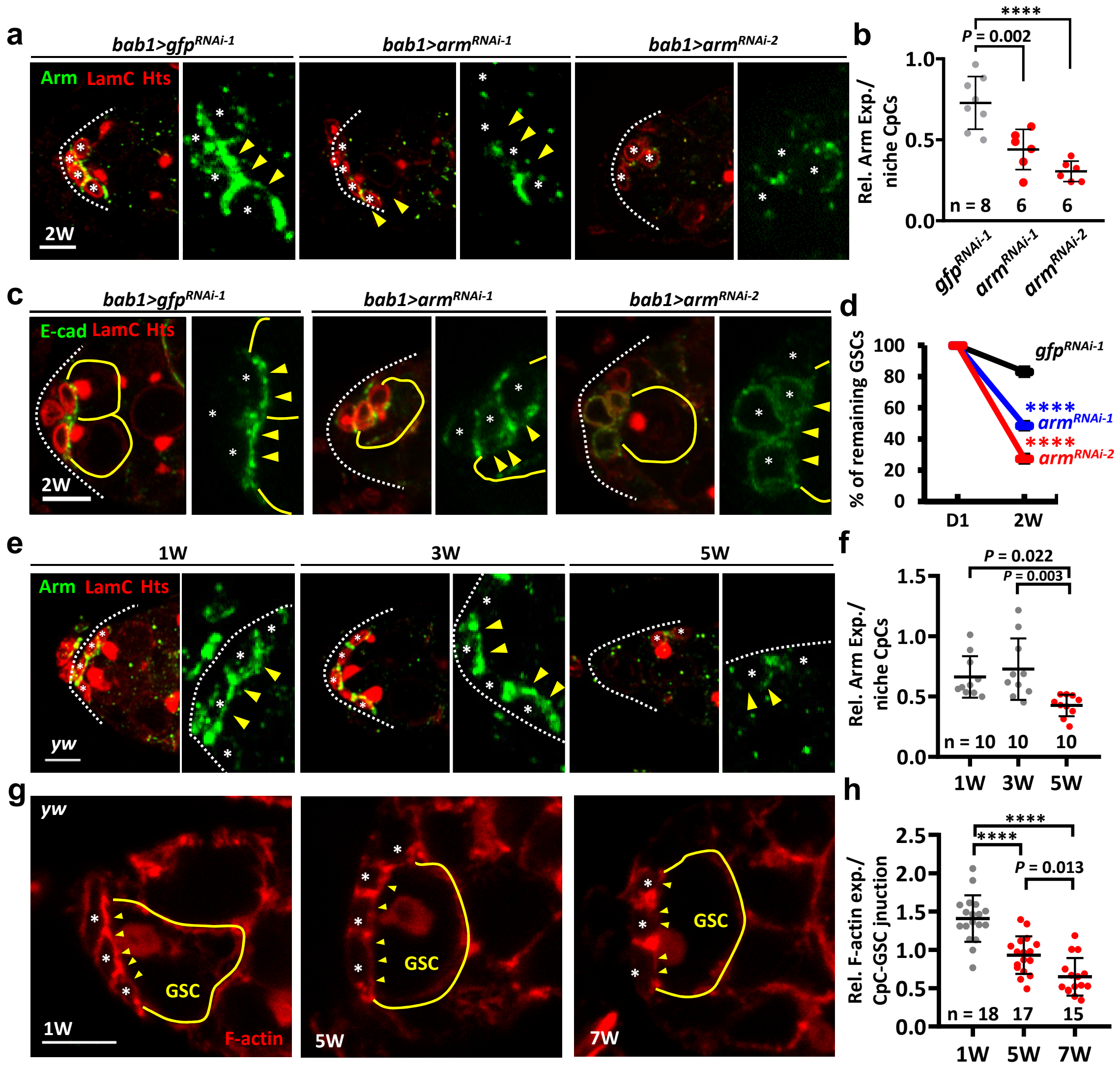

Supplementary figure 9. Niche-expressed Armadillo controls GSC anchoring to the niche, and its expression is reduced in aged niches along with F-actin reduction.

Supplementary Figure 9. Niche-expressed Armadillo controls GSC anchoring to the niche, and its expression is reduced along with decreased F-actin levels in aged niches. a, Armadillo (Arm) expression is reduced in *arm*-KD CpCs (green, Arm; red, LamC for CpCs and Hts for fusomes). Enlargements of the Arm channel are shown next to merged images. **b**, Average (Avg.) expression of Arm per niche in two different arm^{RNAi} lines. **c,** Expression of E-cadherin (E-cad) (green; red, LamC and Hts) is reduced in niche-GSC junction of *arm*-knockdown germaria. The defective E-cad pattern is more severe in $arm^{RNAi\text{-}2}$ line due to its high efficiency of Arm knockdown. **d,** Depletion of niche Arm expression causes GSC loss in a dose-dependent manner. Percentage (%) of remaining GSCs in $bab1{>}gfp^{RNAi}$ (one day(D1), n=120; two-week (2W), n=108), $bab1{>}arm^{RNAi\text{-}1}$ (D1, n=120; 2W, n=102), and $bab1{>}arm^{RNAi\text{-}2}$ (D1, n=120; 2W, n=101) germaria at one day (D1) and two weeks after eclosion. **e**, Armadillo (Arm) expression is reduced in 5-week (W)-old CpCs and the CpC-GSC junction (green, Arm; red, LamC for CpCs and Hts for fusomes). Enlargements of the Arm channel are shown next to merged images. **f**, Average (Avg.) expression of Arm per niche at indicated age. **g and h**, F-actin expression in the CpC-GSC junction of *yw* flies at indicated age. (a, c and e), Yellow arrowheads point to the junctions between GSCs and CpCs; asterisks mark CpCs; white dashed lines mark the anterior edge of the germarium. (b), Yellow lines outline GSCs. Note that GSC number for $bab1{>}gfp^{RNAi}$ are the same data as shown in fig. 1d. Analyzed niche (CpCs) numbers (n) from biologically independent samples are showed in b, f and h). Scale bar (a, c, e and g), 5 μm. Student's *t*-test was used for statistical comparisons in (c). One-way ANOVA was used for statistical analysis in (b, f and h). ****$P < 0.0001$. Data are presented as mean values +/- S.D. (b, e and h) or S.E.M. (d).

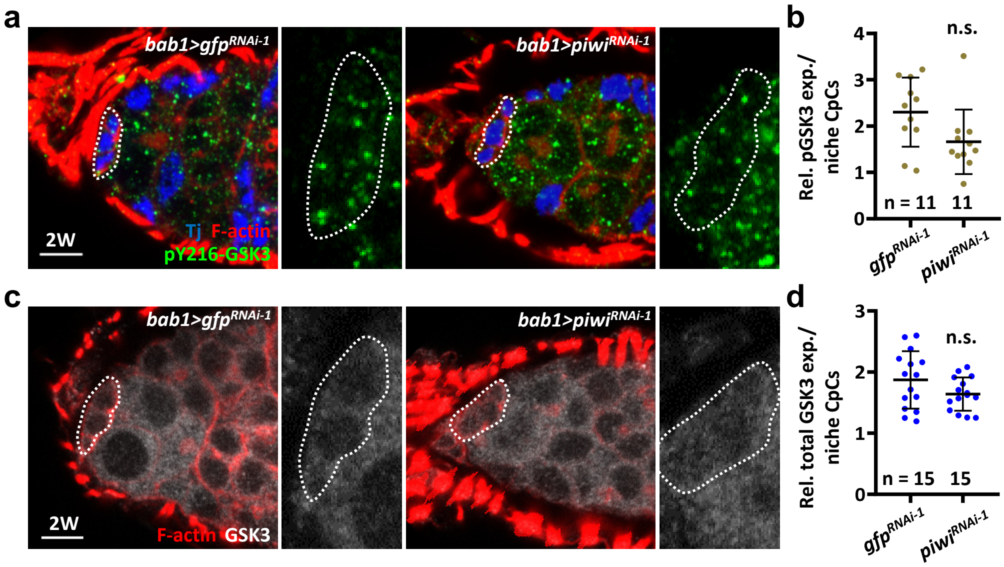

Supplementary Figure 10. pY216-GSK3 and total GSK3 expression are not increased in the *piwi*-KD niche. a, pY216-GSK3 expression in CpCs of indicated genotypes (green, pY216-GSK3; red, F-actin; blue, Tj). White dashed circle, CpCs. **b**, Relative expression of pY216-GSK3 in the niche. **c,** total GSK3 expression in CpCs of indicated genotypes (white, total GSK3; red, F-actin). White dashed circle, CpCs. **d**, Relative expression of total GSK3 in the niche. Data are presented as mean values +/- S.D.. Analyzed niche (CpCs) numbers (n) from biologically independent samples are showed in the graph (a' and b'). Student's *t*-test was used for statistical analysis. n.s., non-significant. Scale bar for a and b, 5μm.

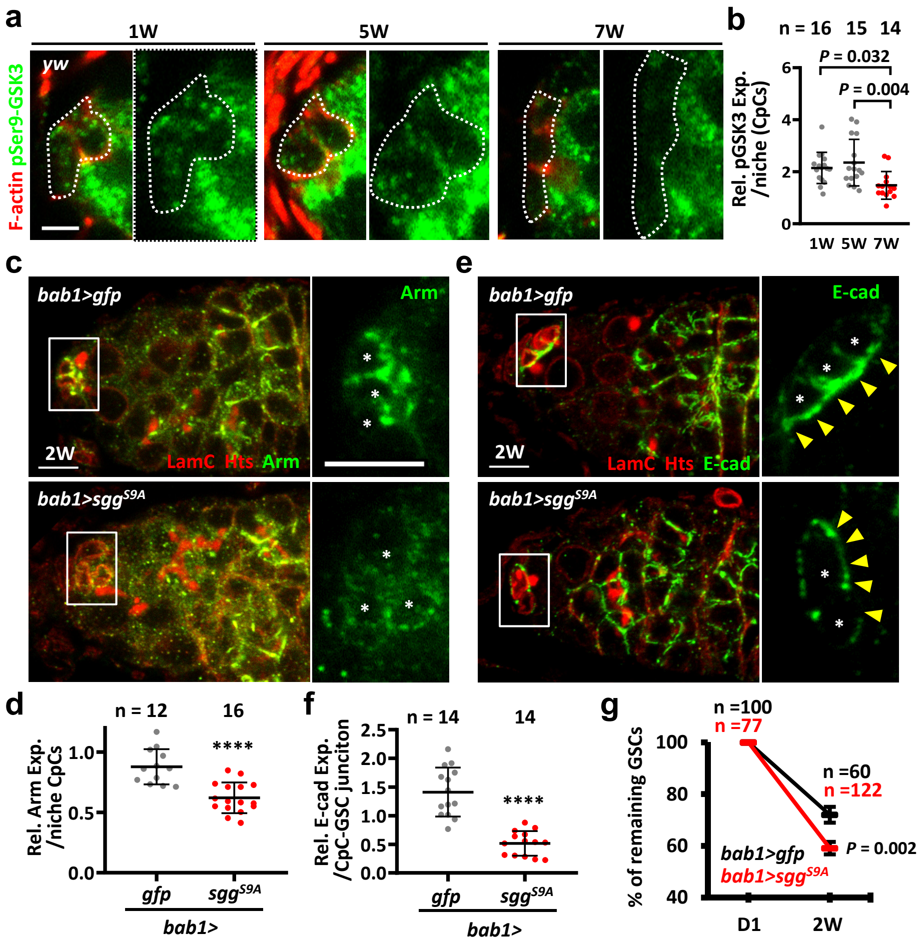

Supplementary Figure 11. Active GSK3 (pSer9-GSK3) level is reduced in aged niche, and overexpressing constitutively active GSK3 (Sgg^{S9A}) in the young niche decreases Arm, E-cad expression and causes GSC loss. a and b, pSer9-GSK3 expression (green; F-actin, red) in CpCs of *yw* flies at indicated age. pSer9-GSK3 levels remained unchanged until 5 weeks of age, but its expression is reduced in 7-week-old flies. Thus, at 5 weeks of age, pGSK3 may be negatively/positively regulated by other signaling pathways in a Piwi-independent but aging-dependent process. **c and d**, Arm expression is reduced in CpCs of *bab1>sgg^{S9A}* flies (green, Arm; LamC and Hts, red). **e and f**, E-cad expression is reduced in the CpC-GSC junction of *bab1>sgg^{S9A}* flies (green, E-cad; red, LamC and Hts). **g,** GSC maintenance is reduced in *bab1>sgg^{S9A}* flies at 2 weeks of age compared to *bab1>gfp* flies. White dashed circles, CpCs; yellow arrowheads point to the junctions between GSCs and CpCs; asterisks mark CpCs. Scale bar, 5 μm. Analyzed sample numbers (n) from biologically independent samples are showed above the graph b, d, f and g. Student's *t*-test was used for statistical analysis in (d, e and g). One-way ANOVA was used for statistical analysis in (b). $****P < 0.0001$. Data are presented as mean values +/- S.D. (b, d and f) or S.E.M. (g).

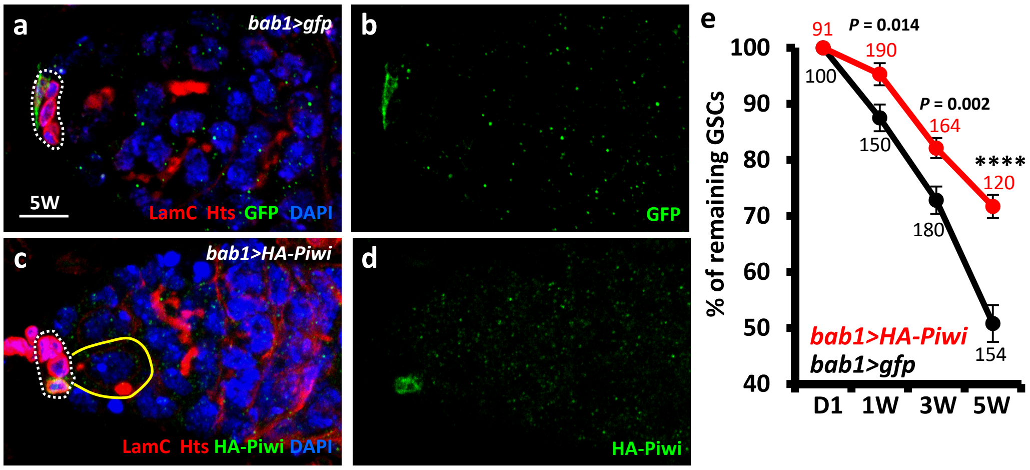

Supplementary Figure 12. Overexpression of Piwi in the niche after eclosion slows age-dependent GSC loss. a-d, Expression of GFP (a, b) and HA-Piwi (c and d) in the 5 week-old aged niche of *bab1>gfp* and *bab1>HA-Piwi* flies (green, GFP and HA-Piwi; Red, LamC and Hts; blue, DAPI) **e,** GSC maintenance is increased in *bab1>HA-Piwi* germaria at indicated age compared to *bab1>gfp* germaria. The number of germaria for each genotype and age are shown above or below the % of remaining GSC. Student's *t*-test was used for statistical analysis to compare GSC maintenance rate of genotypes at the same age. ****$P < 0.0001$. Data are presented as mean values +/- S.E.M.. Scale bar, 5 µm.

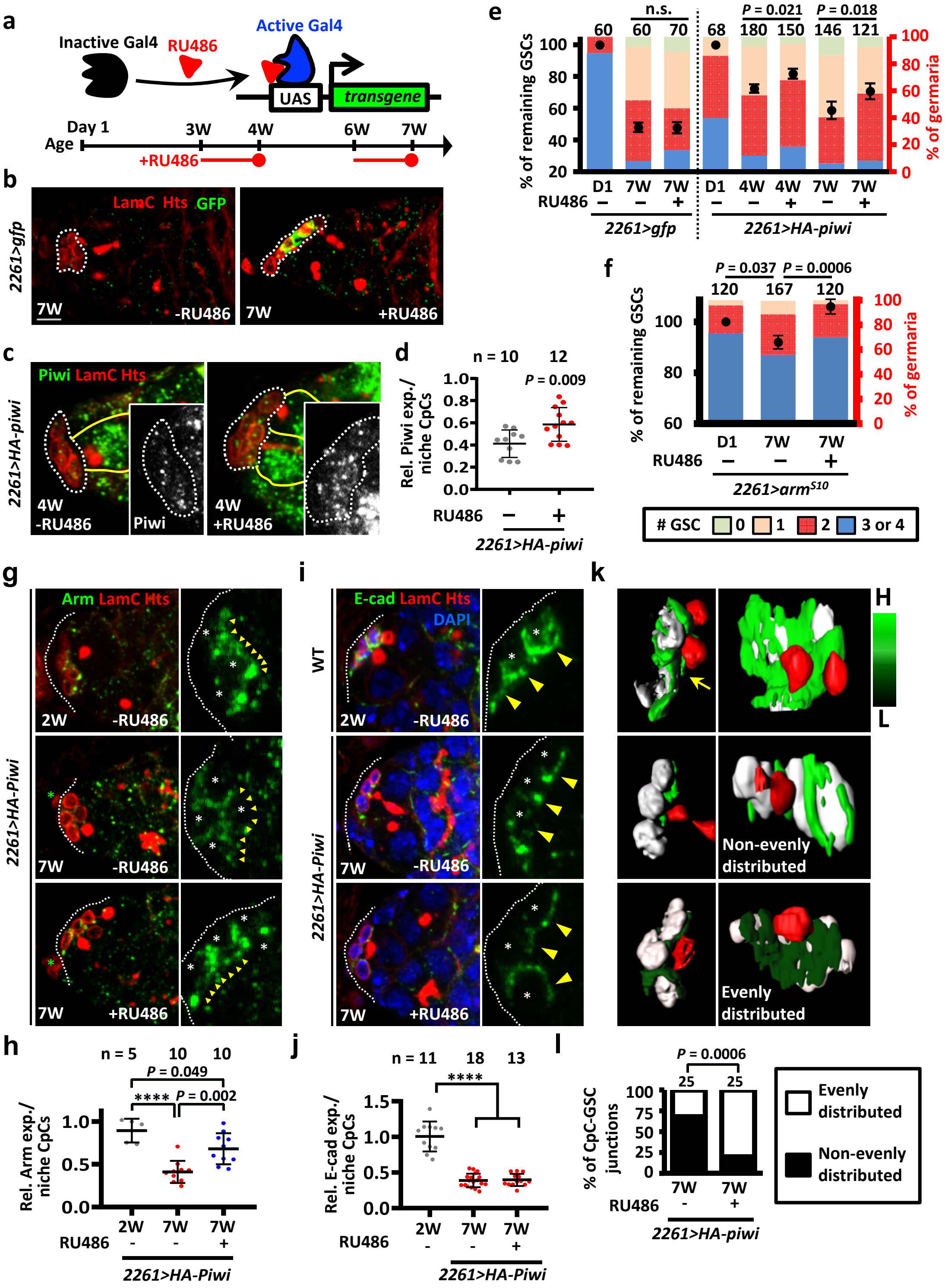

Supplementary figure 13. Piwi supplementation in the aged niche improves Armadillo expression, E-cadherin membrane distribution and GSC maintenance.

Supplementary Figure 13. Piwi supplementation in the aged niche improves Armadillo expression, E-cadherin membrane distribution and GSC maintenance. a, The schematic shows activation of inducible GeneSwitch GAL4 to drive *UAS* transgene expression in flies from 3-4 and 6-7 weeks (W) after ecolsion. GeneSwitch GAL4 is a progesterone receptor-GAL4 fusion protein that is activated by binding RU486 (progesterone analogue). **b**, GFP expression in cap cells (CpCs) of 7-week-old *GeneSwitch GAL4 (#2261)>gfp* flies with (+RU486, 3.6 mM) or without RU486 treatment (-RU486) (green, GFP; red, LamC for CpC nuclei and Hts for fusomes). **c**, #2261 drives HA-Piwi expression in CpCs of 4-week-old flies fed with RU486 (green, Piwi; red, LamC and Hts). Insets show enlarged views of CpCs with Piwi channel (gray) alone. **d**, Relative Piwi expression per niche of *2261>HA-piwi* flies with or without RU486. **e** and **f**, GSC number in *#2261>gfp*, *#2261>HA-piwi* (e), and #*2261>arm*^{S10} (f) flies at indicated ages, with or without RU486 feeding. Note that the reduction of GSCs in #*2261>arm*^{S10} is obviously less than that observed in *#2261>gfp*. This attenuated effect might be due to the known leaky expression of the GeneSwitch system, even without RU486 treatment. This result also indicates that Arm plays a critical role in GSC maintenance, in agreement with the severe loss of GSCs in the *arm*-KD niche (Supplementary Fig. 7). Left Y axis, percentage (%) of remaining GSCs at the indicated age compared with GSC number at Day (D) 1. Right Y axis, % of germaria carrying 0, 1, 2, 3 or 4 GSCs. **g**, Armadillo (Arm) expression of *#2261>HA-piwi* fly is significantly increased in the niche of 7-week-old flies fed with RU486 (green, Arm; red, LamC and Hts). **h**, Relative Arm expression per niche of *2261>HA-piwi* flies at indicated age, with or without RU486. **i**, E-cadherin (E-cad) distribution in the niche-GSC junction, but not expression level, is rescued by RU486 treatment in 7-week-old #*2261>HA-piwi* flies (green, E-cad; red, LamC and Hts). **j,** Relative E-cadherin expression in the niche of #*2261>HA-piwi* flies with or without RU486, at indicated age. **k**, E-cad distribution is rescued in #*2261>HA-piwi* fly upon RU486 treatment. 3D reconstruction of contact area between CpCs (gray) and GSCs (indicated by fusomes, red) reveals E-cadherin distribution (green) and expression levels in 2-week-old wide-type and 7-week-old #*2261>HA-piwi* females with or without RU486 treatment. The green gradient (high[H] to low[L]) indicates strength of E-cadherin expression. **l**, % of niche-GSC junctions with evenly or non-evenly distributed E-cadherin expression in 7-week-old #*2261>HA-piwi* females with or without RU486 treatment. Dashed circles outline the niche (b, c); Dashed lines mark the anterior edge of the germarium (g, i); asterisks mark CpCs (g, i); arrowheads indicate the niche-GSC junction (g, i); arrow, the direction of CpC-GSC contact area visualization (k). Scale bars, 5 μm. The numbers of analyzed niches or GSCs are shown within or above each bar graph (e, f and l) or dot plots graph (d, h and j). Data are presented as mean values +/- SD (d, h and i) or S.E.M. (e and f). Student's *t*-test (d, e, f), chi-squared test (l) and One-way ANOVA (h and j) were used for statistical analyses. ****$P < 0.0001$. n.s., non-significant.

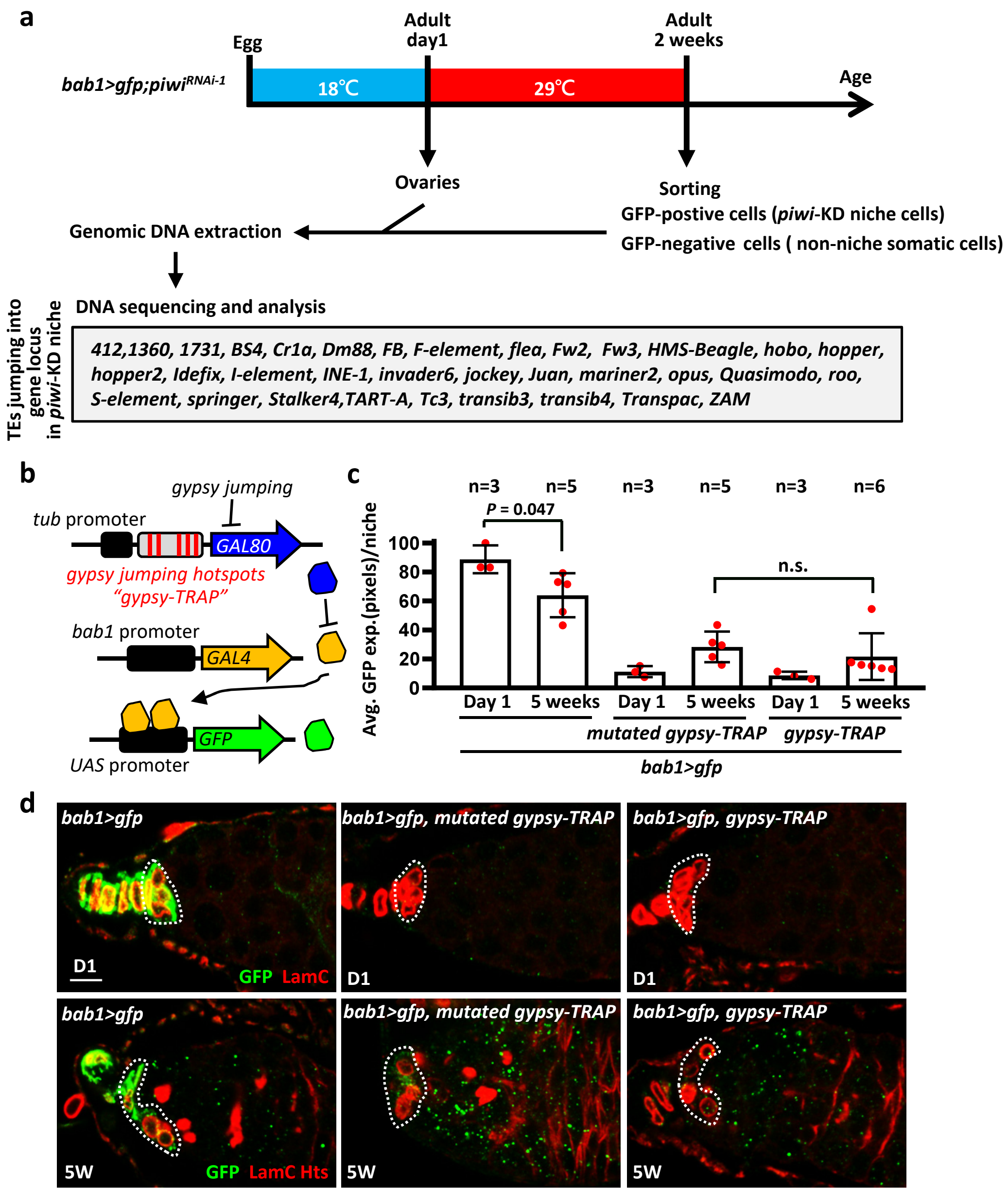

Supplementary figure 14. ***gypsy*** **retrotransposon dose not have new retrotransposition in in the** ***piwi*****-KD and aged CpCs**

Supplementary Figure 14. *gypsy* retrotransposon does not exhibit new retrotransposition in *piwi*-KD and aged CpCs. a, Flowchart showing isolation of *piwi*-KD niche cells (CpC and TFs) for whole genomic DNA sequencing. *bab1> gfp;piwi*$^{RNAi\text{-}1}$ *flies* were grown at 18°C to silence GAL4 activity and switched to 29°C after eclosion to knock down Piwi. Genome of ovaries from newly eclosed *bab1> gfp;piwi*$^{RNAi\text{-}1}$ flies (Day 1) served as a genotype control. GFP-positive *piwi*-KD niche cells (CpCs and TFs) and GFP-negative ovarian non-niche somatic cells (such as ECs and FCs, which served as another control for new transposon insertions) were isolated from ovaries of 2-week-old *bab1>gfp;piwi*^{RNAi} flies. TEs listed in the rectangle show *de novo* insertion sites in or near the gene locus in the genome of the *piwi*-KD niche cells. **b,** The *gypsy*-TRAP system. The *ovo* regulatory region, which is targeted by *gypsy* (~500 base pairs, *gypsy* trapping sequence), is placed between a tubulin promoter and the coding sequence of GAL80. GAL80 is produced and silences activity of GAL4 driven by the *bab1* promoter in CpCs. Upon *gypsy* insertion into the *ovo* regulatory region, GAL80 production is disrupted, and *bab1-GAL4* drives GFP expression in cap cells. **c** and **d**, *gypsy* is not trapped by the *gypsy*-TRAP system in aged CpCs. *bab1>gfp* flies express GFP (green) in CpCs (red, LamC) of one-day-old (D1) or 5-week (W)-old flies. GFP expression (green; red, LamC and Hts) is not observed in one-day-old (D1) or 5-week (W)-old flies carrying the *gypsy*-TRAP system, or the mutated *gypsy*-TRAP system, in which *ovo* regulatory sequence is mutated and cannot trap TEs. Hts mark fusomes. The numbers of analyzed niches are shown within or above each bar. Data are presented as mean values +/- SD (c). Student's *t*-test was used for statistical analysis; n.s., non-significant. Scale bar in d, 5 μm.

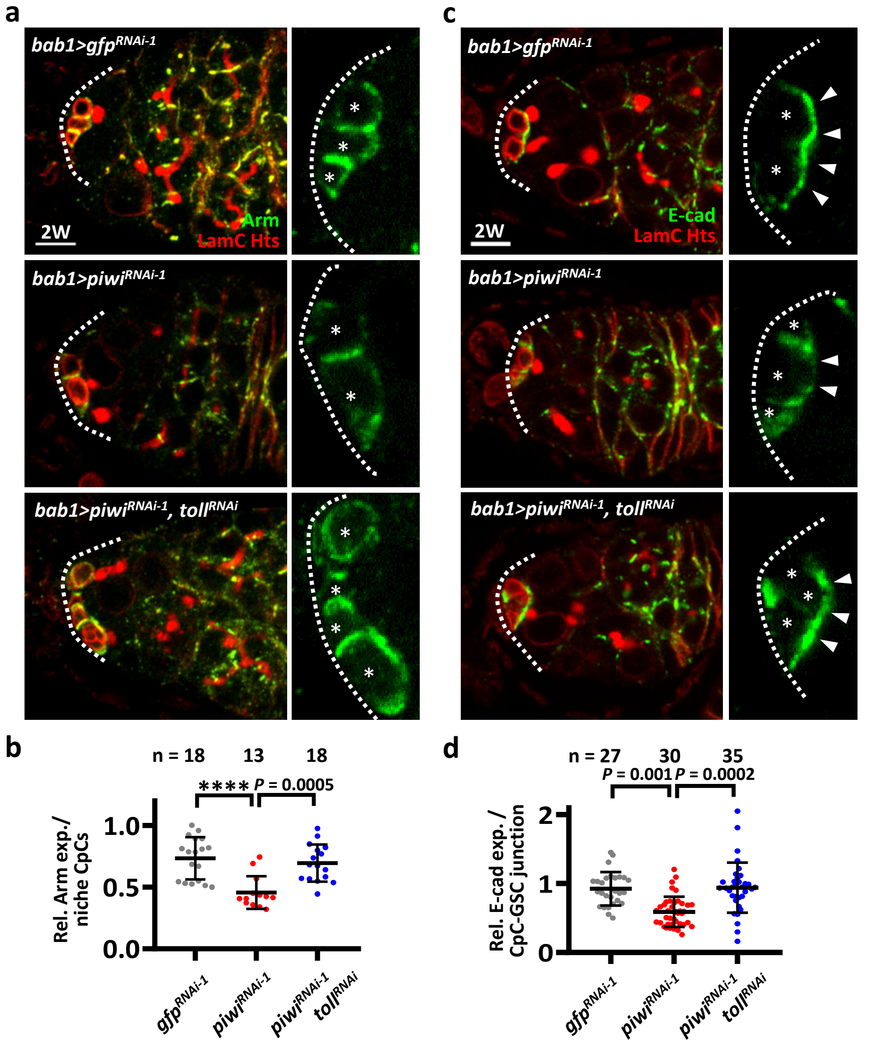

Supplementary Figure 15. Depletion of Toll rescues E-cad membrane localization and Arm expression in the *piwi*-KD CpCs. a, Arm expression in the niche of 2-week-old *bab1>gfp*$^{RNAi\text{-}1}$, *bab1>piwi*$^{RNAi\text{-}1}$ and *bab1>piwi*$^{RNAi\text{-}1}$;*toll*^{RNAi} flies (green, Arm; red, LamC for CpC nuclear envelopes; red, Hts for fusomes). **b,** Relative expression of Arm in the niche of the germarium with indicated genotypes. **c,** E-cadherin expression in the niche-GSC junction of 2-week-old *bab1>gfp*$^{RNAi\text{-}1}$, *bab1>piwi*$^{RNAi\text{-}1}$ and *bab1>piwi*$^{RNAi\text{-}1}$;*toll*^{RNAi} flies (green, E-cad; red, LamC and Hts). Arrowheads, junction of niche-GSC. **d,** Relative expression of E-cadherin in the niche-GSC junctions of germaria with the indicated genotypes. The numbers of analyzed niches or niche-GSC junction are shown above the graph (b and d). One-way ANOVA was used for statistical analysis (b and d). ****$P < 0.0001$. Data are presented as mean values +/- SD (b and d). Scale bars, 5 μm.

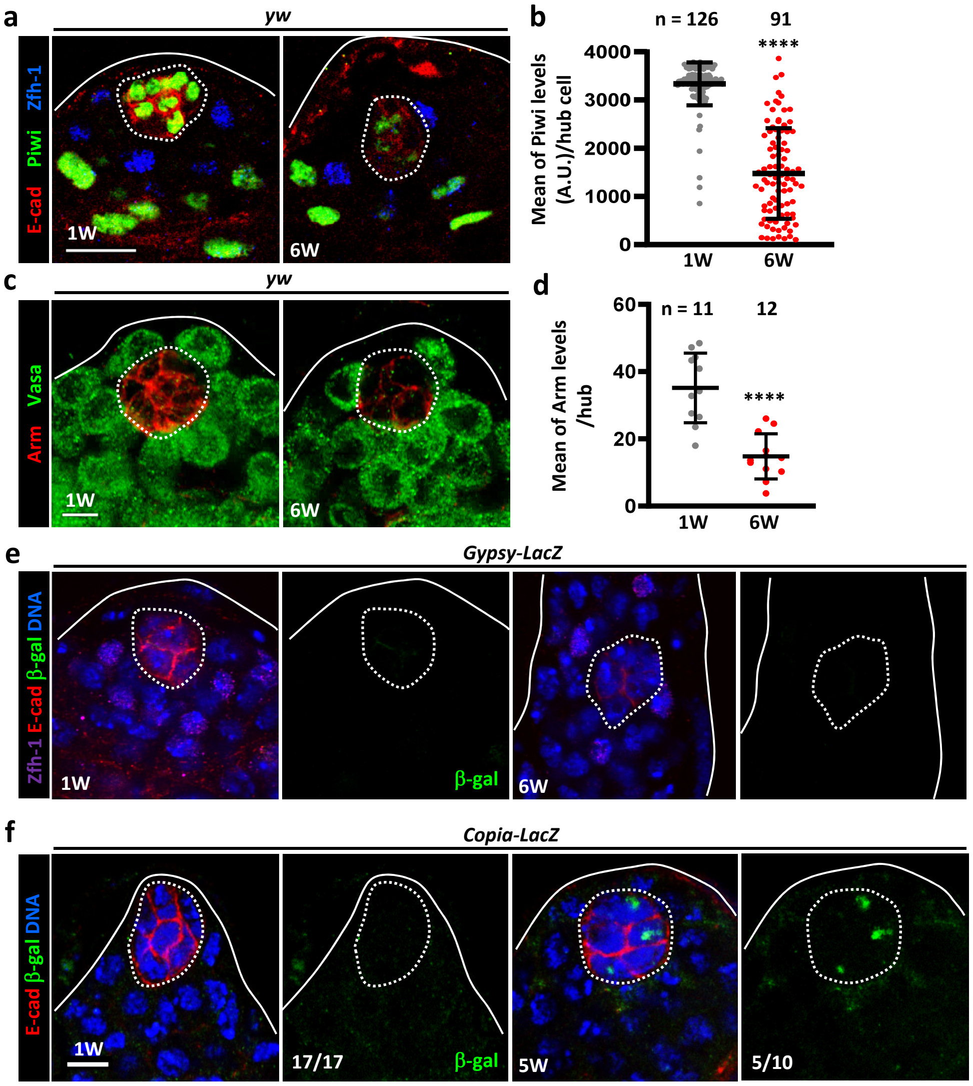

Supplementary Figure 16. Piwi and Armadillo expression levels are reduced while expression of *copia-lacZ* is increased in the aged male GSC niche. a, Piwi expression (green; red, E-cadherin (E-cad); blue, Zfh-1 for somatic cysts) is reduced in 6-week (W)-old niche hub cells (green, Piwi; red, E-cadherin (E-cad); blue, Zfh-1 for somatic cysts). **c**, Armadillo (Arm) expression is decreased in 6-week (W)-old niche hub cells (red, Arm; green, Vasa for germ cells). **b** and **d,** Average mean intensity (A.U.) of Piwi (b) and Arm expression (d) in 1- and 6-week-old hub cells. The numbers of analyzed hub cells are shown in each bar. Data are presented as mean values +/- SD. Student's *t*-test was used for statistical analysis. ****P < 0.0001. **e**, *gypsy-LacZ* is not expressed in aged hub cells (green, b-gal for LacZ; red, E-cad; blue, DAPI for DNA; purple, Zfh-1). **f**, *copia-LacZ* expression is increased in aged hub cells (green, b-gal for LacZ; red, E-cad; blue, DAPI for DNA). Dashed circles outline hub cells; white line indicates the edge of anterior testis. Scale bar, 5 μm. A.U., arbitrary units.

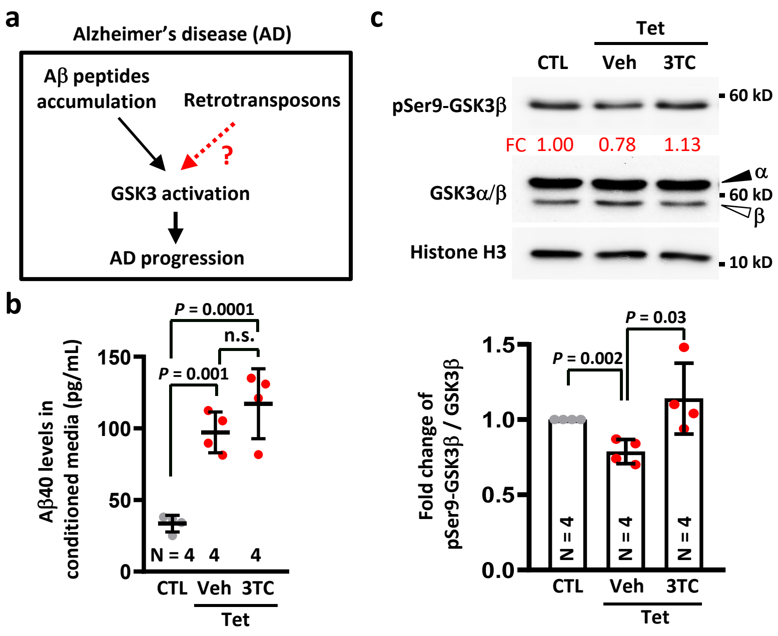

Supplementary Figure 17. 3TC treatment suppresses GSK3 activity in human cell model of Alzheimer's disease (AD). a, Abnormal Ab peptide accumulation promotes GSK3 activity contributing to AD progression, but the mechanisms of this action are still unclear. Recently, retrotransposon activation has been reported in AD patients, raising the possibility that GSK3 activation might be due to retrotransposon activity. **b,** Tetracycline (Tet) treatment for 72 hours induces Aβ40 production in human embryonic kidney (HEK) 293-derived CG cells, which express APP-C99. The APP-C99 protein is cleaved by γ-secretase to release Aβ peptides. **c,** Expression level of pSer-GSK3 is reduced in CG cells with Ab accumulation, and 3TC treatment restores this effect. CTL, control. Veh, vehicle (water). FC, fold change. Black arrowhead indicates GSK3a (a), while white arrowhead indicates GSK3β (b). Data showing pSer9-GSK3 and Ab levels were from four independent experiments (N = 4). kD, kiloDalton. Data are presented as mean values +/- SD (b and c). One-way ANOVA (b) and Student's *t*-test (c) were used for statistical analysis. n.s., non-significant.

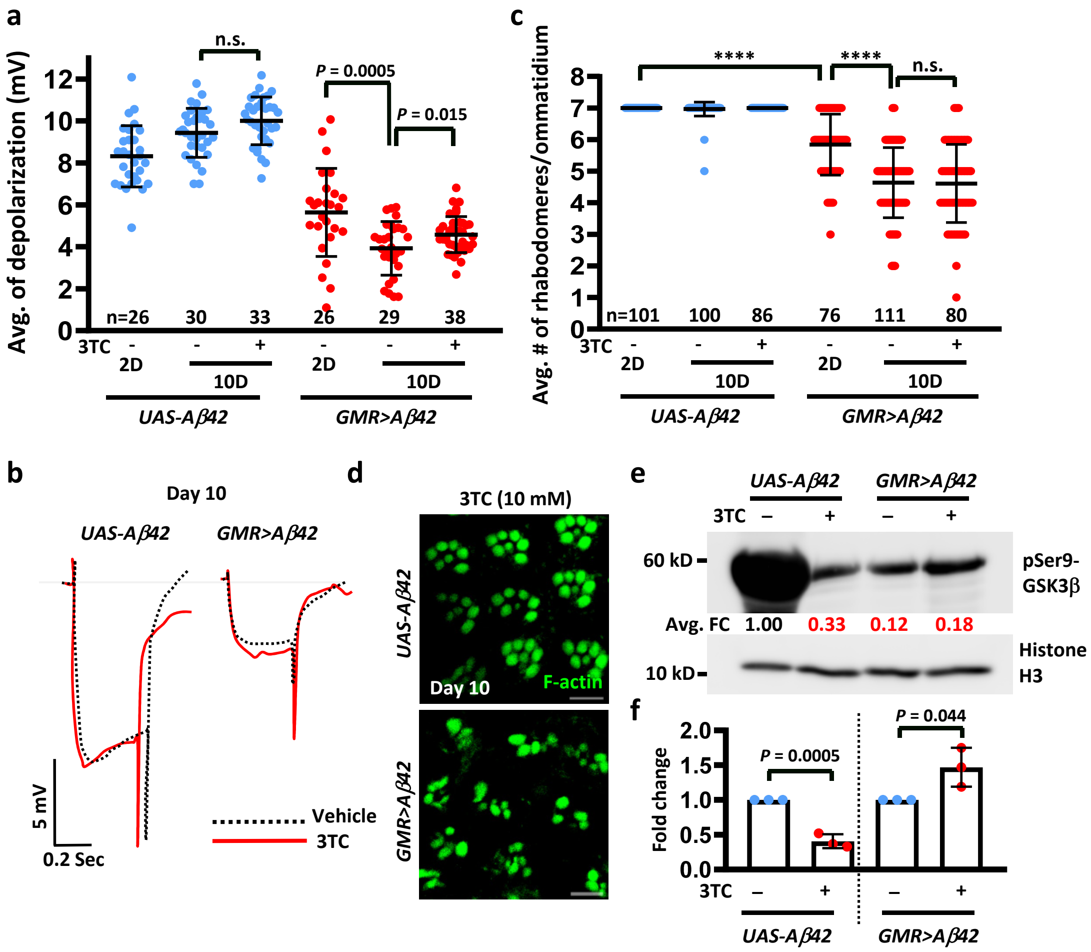

Supplementary Figure 18. 3TC treatment partially suppresses GSK3 activity induced in the Aβ42-overexpressing fly eyes. a, Depolarization amplitudes in Electroretinogram (ERG) responses to light exposure, as an indication of photoreceptor activity. Newly eclosed flies with eye-specific *Aβ42* expression (*GMR>Aβ42*) were treated with or without 3TC (10 mM), and ERG responses were recorded at day 2 (2D) or day (10D). **b,** Representative ERG traces at day 10 for indicated genotypes. **c,** Quantification of rhabodomeres per ommatidium in the indicated genotypes with or without 3TC treatment. **d,** Representative cross-sections of the eye at day 10 for indicated genotypes, showing rhabdomeres in green (Phalloidin). Scale bar, 5mm. **e**, pSer9-GSK3 levels in the eyes of 10-day-old *UAS-Aβ42/+* and *GMR>Aβ42* flies with or without 3TC treatment. Average fold change (Avg. FC) of pSer9-GSK3 expression levels in the eyes of indicated group were normalized to *UAS-Aβ42/+* eyes without 3TC treatment. Note that pSer9-GSK3 expression is extremely highly in *UAS-Ab42/+* eyes and 3TC-treated flies display remarkable reduction of pSer9-GSK3 expression, probably due to the genotype. **f,** Average fold change of pSer9-GSK3 expression levels in the eyes of indicated genotypes with or without 3TC treatment from three independent experiments (N = 3). kD, kiloDalton. Student's *t*-test (a, c and f) was used for statistical analysis. $****P < 0.0001$. n.s., non-significant. Data are presented as mean values +/- SD.

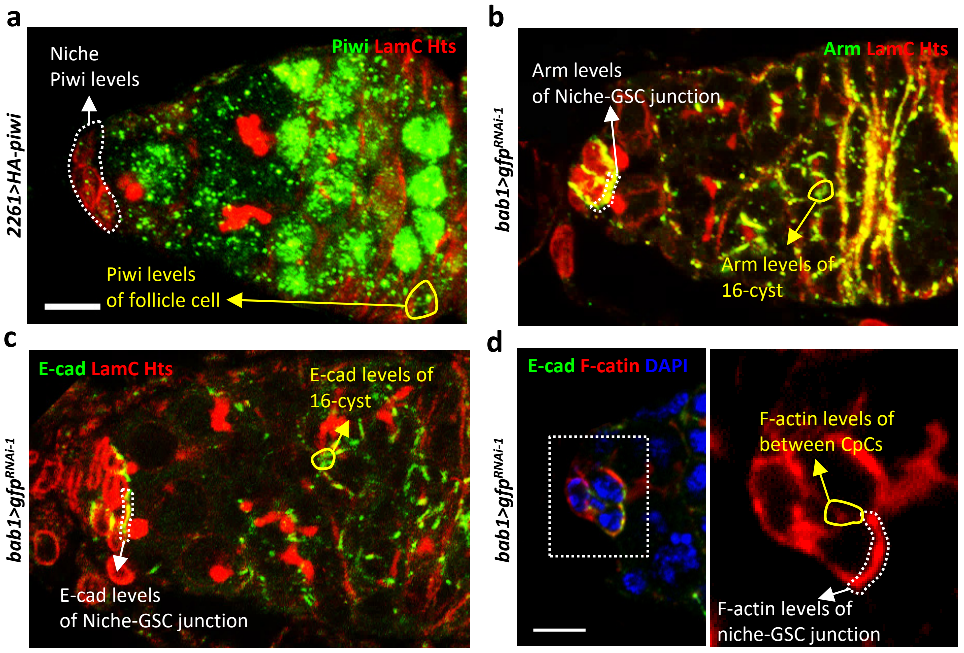

Supplementary Figure 19. Measurement of protein expression in the niche CpCs. **a**, Quantification of Piwi expression in the niche of *2261>HA-piwi* fly. Piwi expression in the niche (marked by the white dashed circles) was normalized to Piwi expression in follicle cells (marked by the yellow circle) to obtain relative Piwi expression (green, Piwi; red, LamC for CpC nuclear envelopes and Hts for fusomes). **b**, Quantification of Armadillo (Arm) expression in the niche-GSC junction. Arm expression in the niche-GSC junction (indicated by the white dashed circle) was normalized to Arm expression in one germ cell from a 16-cell cyst (marked by a yellow circle) to obtain relative Arm expression (green, Arm; red, LamC and Hts). **c**, Quantification of E-cadherin (E-cad) expression in the niche-GSC junction. E-cad expression in the niche-GSC junction (marked by the white dashed circle) was normalized to E-cad expression in one germ cells from a16-cell cyst (marked by the yellow circle) to obtain relative E-cad expression (green, E-cad; red, LamC and Hts). **d**, Quantification of F-actin expression. F-actin expression in the niche-GSC junction (indicated by the white dashed circle) was normalized to F-actin expression between two cap cells (yellow circle) to obtain relative F-actin expression (red, F-actin; green, E-cad; blue, DAPI). The square region with niche cells in the left image is enlarged in the right image and only shows F-actin signal. The genotype in b-d is *bab1>gfp*$^{RNAi-1}$. Scale bar (a and d), 5 mm. Images (a, b and c) are the same magnification and share scale bar with a.

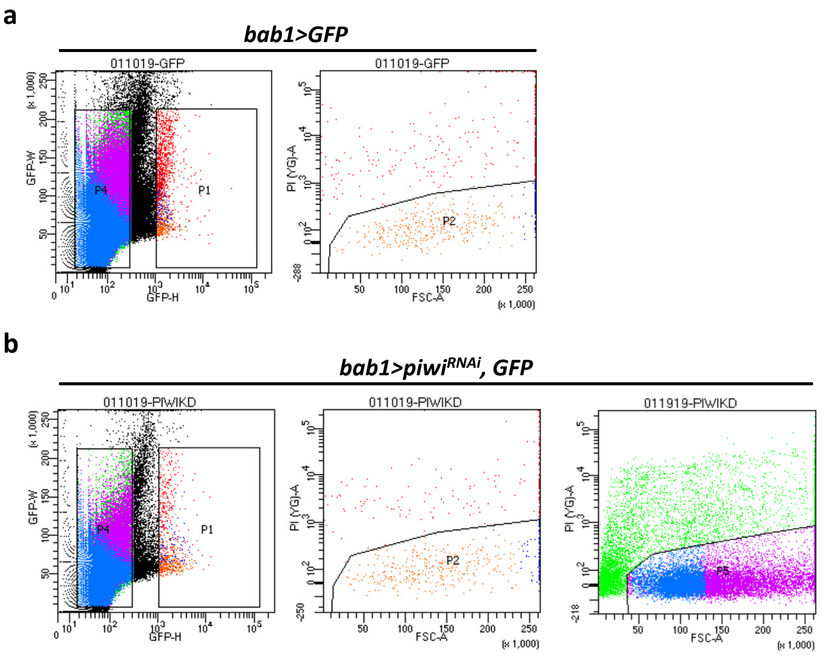

Supplementary figure 20. Gate strategy for isolation of niche cells. a, A example of Gating strategy to sort GFP-positive niche cells of *bab1>GFP* (a) and *bab1>piwi*^{RNAi}*, GFP* ovary. GFP-positive cells from Panel 1 (P1) were selected and cells with low PI intensity (Panel 2), stand for non-dead cells, were sorted and subjected to RNA extraction and RNA-sequencing. The results were used on the analysis of transposon expression profile. **b,** A example of Gating strategy to sort GFP-positive niche cells and GFP-negative non-niche cells of *bab1>piwi*^{RNAi}*, GFP* ovary. GFP-positive niche cells from Panel 1 (P1) were selected and cells with low PI intensity (P2) were further sorted, while GFP-negative non-niche cells from P4 were selected and cells with low PI intensity (P5) were further sorted, sorted cells were subjected to DNA extraction and whole genomic sequencing and the results were used on the analysis of novel retrotransoposition of transposon in the *piwi*-knockdown niche.

Supplementary table 1. Numbers of GSCs in germaria of indicated genotypes at different ages

Genotypes	[§]time after eclosion/ [#]percentage of germaria/ [¥]average number of GSCs / [¶]percentage of remaining GSCs											
	[§]Day 1				2 weeks				5 weeks			
	≥3 GSCs	2 GSCs	1 GSC	0 GSC	≥3 GSCs	2 GSCs	1 GSC	0 GSC	≥3 GSCs	2 GSCs	1 GSC	0 GSC
bab1>gfp^{RNAi-1}	[#]46	53	1	0	26	54	18	2	12	51	31	6
	[¥]2.5 ± 0.6 (n=120)				2.1 ± 0.8 (n=108)				1.7 ± 0.8 (n=120)			
	[¶]100 ± 2.1				83 ± 2.9				68 ± 2.8			
[Δ]*bab1>gfp*$^{RNAi-2}$	44	50	6	0	26	59	13	2	9	46	42	3
	2.4 ± 0.6 (n=120)				2.1 ± 0.7 (n=120)				1.6 ± 0.7 (n=120)			
	100 ± 2.3				88 ± 2.6				68 ± 2.8			
bab1>piwi^{RNAi-1}	47	48	5	0	14	42	39	5	1	24	54	21
	2.5 ± 0.7 (n=120)				1.6 ± 0.8 (n=94)				1.0 ± 0.7 (n=120)			
	100 ± 2.6				66 ± 3.3***				43 ± 2.6***			
bab1>piwi^{RNAi-2} (for Fig. 1d)	76	23	1	0	28	42	22	8	3	38	47	12
	2.8 ± 0.5 (n=101)				1.9 ± 0.9 (n=101)				1.3 ± 0.8 (n=120)			
	100 ± 1.9				68 ± 3.3***				47 ± 2.4***			
bab1>piwi^{RNAi-2} (for Fig.2e)	N/A				28	41	27	4	N/A			
					1.9 ± 0.8 (n=197)							
					69 ± 2.1***							
bab1>sgg^{RNAi-1}	70	30	0	0	32	60	12	0	N/A			
	2.8 ± 0.6 (n=120)				2.2 ± 0.7 (n=111)							
	100 ± 2.0				78 ± 2.2							
bab1>Toll^{RNAi}	68	31	1	0	48	45	7	0	N/A			
	2.79 ± 0.7 (n=101)				2.44 ± 0.7 (n=110)							
	100 ± 2.2				88 ± 2.3							
bab1>Toll-5^{RNAi}	86	14	0	0	26	54	20	0	N/A			
	3.09 ± 0.6 (n=101)				2.07 ± 0.7 (n=120)							
	100 ± 1.9				67 ± 2.1***							
bab1>Toll-7^{RNAi}	71	29	0	0	27	62	10	1	N/A			
	2.78 ± 0.6 (n=103)				2.16 ± 0.6 (n=120)							
	100 ± 2.0				78 ± 2.1							
bab1>gfp *(D1, 1W and 2W)*	46	53	1	0	60	35	5	0	30	55	15	0
	3.05 ± 0.7 (n=100) (D1)				2.67 ± 0.7 (n=150) (1W)				2.18 ± 0.7 (n=60) (2W)			
	100 ± 2.1				87 ± 5.9				72 ± 3.1			
bab1>gfp *(3W and 5W)*	34	52	13	1	10	42	40	8	N/A			
	2.22 ± 0.7 (n=180) (3W)				1.55 ± 0.8 (n=154) (5W)							
	73 ± 1.7				51 ± 2.1							
bab1>sgg^{S9A}	78	21	1	0	12	49	34	5	N/A			
	2.87 ± 0.6 (n=77)				1.69 ± 0.8 (n=122)							
	100 ± 2.3				59 ± 2.4**							
bab1>HA-Piwi *(D1, 1W and 2W)*	19	74	7	0	19	61	19	1	13	57	28	2
	2.12 ± 0.5 (n=91) (D1)				2.12 ± 0.5 (n=91) (1W)				1.82 ± 0.7 (n=60) (2W)			
	100 ± 2.4				98 ± 2.3*				86 ± 4.1			
bab1>HA-Piwi *(3W and 5W)*	9	57	32	2	9	40	44	7				
	1.74 ± 0.6 (n=164) (3W)				1.52 ± 0.7 (n=120) (5W)							
	82 ± 2.4**				72 ± 3.2***							
bab1>gfp^{RNAi-1} w/o 3TC	N/A				26	52	22	0	N/A			
					2.1 ± 0.7 (n=100)							
					82 ± 2.9							
bab1>gfp^{RNAi-1} with 3TC	N/A				19	63	18	0	N/A			
					2.0 ± 0.6 (n=100)							
					80 ± 2.4							
bab1>piwi^{RNAi-1} w/o 3TC	N/A				17	42	34	7	N/A			
					1.7 ± 0.8 (n=100)							
					67 ± 3.3							
bab1>piwi^{RNAi-1} with 3TC	N/A				26	53	21	0	N/A			
					2.0 ± 0.8 (n=100)							
					85 ± 3.3***							
bab1>piwi^{RNAi-2} w/o 3TC	N/A				26	53	21	0	N/A			
					2.1 ± 0.7 (n=234)							
					74 ± 1.7							

bab1>piwi^{RNAi-2} with 3TC		N/A			26	53	21	0	N/A
						2.3 ± 0.7 (n=216)			
						82 ± 1.7***			
bab1>piwi^{RNAi-1}, *gfp*$^{RNAi-2}$	69	30	1	0	19	46	32	3	N/A
		2.7 ± 0.6 (n=80)				1.8 ± 0.8 (n=210)			
		100 ± 2.3				67 ± 2.0			
bab1>piwi^{RNAi-2}, *gypsy*^{RNAi}	42	50	7	1	19	54	27	0	N/A
		2.3 ± 0.7 (n=199)				1.9 ± 0.7 (n=125)			
		100 ± 1.9				82 ± 2.6**			
bab1>piwi^{RNAi-2}, *copia*^{RNAi}	80	20	0	0	36	46	16	2	N/A
		3.1 ± 0.8 (n=142)				2.2 ± 0.8 (n=182)			
		100 ± 2.1				71 ±1.9			
bab1>piwi^{RNAi-2}, *loki*^{RNAi}	58	39	3	0	13	48	36	3	N/A
		2.6 ± 0.6 (n=117)				1.7 ± 0.7 (n=122)			
		100 ± 2.3				65 ± 2.5			
bab1>piwi^{RNAi-1}, *p53*$^{-/+}$	61	37	2	0	12	33	40	15	N/A
		2.7 ± 0.6 (n=120)				1.4 ± 0.9 (n=60)			
		100 ± 2.2				54 ± 4.4*			
bab1>arm^{RNAi-1}	46	53	1	0	3	38	50	9	N/A
		2.8 ± 0.8 (n=120)				1.4 ± 0.7 (n=102)			
		100 ± 2.6				48 ± 2.2***			
bab1>arm^{RNAi-2}	49	50	1	0	0	10	49	41	N/A
		2.5 ± 0.5 (n=120)				0.7 ± 0.7 (n=101)			
		100 ± 1.9				27 ± 2.4***			
bab1>piwi^{RNAi-1}, *sgg*$^{RNAi-1}$	67	30	3	0	36	54	10	0	N/A
		2.8 ± 0.7 (n=120)				2.3 ± 0.7 (n=120)			
		100 ± 2.3				82 ± 3.7***			
$^{\Delta}$*bab1>piwi*$^{RNAi-1}$, *sgg*$^{RNAi-2}$	73	26	1	0	44	46	10	0	N/A
		2.9 ± 0.7 (n=75)				2.4 ± 0.8 (n=72)			
		100 ± 2.8				84 ± 3.1***			
bab1>piwi^{RNAi-1}, *arm*^{S10}	68	31	1	0	42	43	14	1	N/A
		2.8 ± 0.7 (n=120)				2.3 ± 0.8 (n=120)			
		100 ± 2.2				82 ± 2.6***			
bab1>piwi^{RNAi-1}, *toll*^{RNAi}	79	21	0	0	52	41	7	0	N/A
		3.0 ± 0.7 (n=120)				2.4 ± 0.7 (n=147)			
		100 ± 2.1				80 ± 1.9***			
bab1>piwi^{RNAi-1}, *toll-5*^{RNAi}	86	14	0	0	58	34	8	0	N/A
		3.2 ± 0.7 (n=128)				2.6 ± 0.8 (n=161)			
		100 ± 1.8				81 ± 1.8***			
bab1>piwi^{RNAi-1}, *toll-7*^{RNAi}	78	22	0	0	26	50	22	2	N/A
		3.0 ± 0.7 (n=117)				2.0 ± 0.8 (n=123)			
		100 ± 2.0				67 ± 2.3			
ptc>gfp^{RNAi-1}	75	25	0	0	10	58	28	4	N/A
		2.8 ± 0.6 (n=64)				1.7 ± 0.7 (n=109)			
		100 ± 2.4				62 ± 2.3			
ptc>piwi^{RNAi-1}	75	22	19	0	16	52	29	3	N/A
		2.9 ± 0.7 (n=65)				1.8 ± 0.7 (n=102)			
		100 ± 3.2				62 ± 2.5			

Genotypes	Day 1 ≥3 GSCs	Day 1 2 GSCs	Day 1 1 GSC	Day 1 0 GSC	4 weeks ≥3 GSCs	4 weeks 2 GSCs	4 weeks 1 GSC	4 weeks 0 GSC	7 weeks ≥3 GSCs	7 weeks 2 GSCs	7 weeks 1 GSC	7 weeks 0 GSC
2261>gfp	[#]88	12	0	0		N/A			8	45	40	7
		[¥]3.2 ± 0.7 (n=60)								1.6 ± 0.8 (n=60)		
		[¶]100 ± 2.9								48 ± 3.0		
2261>gfp with RU486		N/A				N/A			16	32	41	11
										1.5 ± 1.0 (n=70)		
										48 ± 3.5		
2261>HA-piwi	40	46	13	1	12	44	37	7	6	34	46	14
		2.3 ± 0.8 (n=68)				1.6 ± 0.8 (n=180)				1.3 ± 0.8 (n=146)		
		100 ± 3.4				72 ± 2.6				59 ± 5.5		
2261>HA-piwi with RU486		N/A			19	49	27	5	8	50	35	7
						1.8 ± 0.9 (n=150)				1.6 ± 0.8 (n=121)		
						82 ± 3.1*				71 ± 5.0*		
2261>arm^{S10}	73	23	4	0		N/A			55	33	11	1
		2.9 ± 0.7 (n=120)								2.6 ± 1.0 (n=167)		
		100 ± 2.4								90 ± 2.6		
2261>arm^{S10} with RU486		N/A				N/A			70	27	3	0
										3.0 ± 0.9 (n=120)		
										103 ± 2.9***		

Newly eclosed *bab1>gene*^{RNAi} and *ptc>gene*^{RNAi} flies were grown on standard food at 29°C. Ovaries were dissected at indicated ages, then immunostained and analyzed. Newly eclosed *2261>transgenes* flies were grown on standard food at 25°C. Ovaries were dissected at indicated ages, then immunostained and analyzed. For experiments with 3TC and RU486 treatment, results were statistically analyzed, with comparisons made between non-treated and treated groups of the same genotype at same age. Other statistical comparisons were made between control groups (either *gfp*^{RNAi} or *piwi*$^{RNAi-2}$ or *piwi*$^{RNAi-1}$,*gfp*$^{RNAi-2}$ groups) and experimental groups. Total numbers of analyzed germaria of each genotype at the specific age are shown in parentheses.

[§] Ages after eclosion of dissected flies.

[#] Percentage of germaria carrying ≥3, 2, 1 and 0 GSC(s) at indicated genotypes and age.

[¥] Average number of GSCs with standard deviation (SD) per germarium.

[¶] Percentage of remaining GSCs with standard error of the mean (SEM) relative to day 1 GSC number of the same genotype.

[Δ] Results obtained from RNAi lines are only shown in extended data table 1.

*Significant difference relative to controls: P < 0.05;

** Significant difference relative to controls at same age: P < 0.01;

***Significant difference relative to controls at same age: P < 0.001.

N/A indicates not determined.

Supplementary table 2. New transposon insertions mapped in 2-week-old *piwi* -knockdown niche cells.						
A	B	C	D	E	F	G
Events	Chr.	TE	Insertion classification	Gene name	Attribute	Coverage_Ratio
1	chr2L	*I-element*	intron	*Pvr*	*piwi-* KD1	0.1
2	chr2L	*I-element*	intergenic	*CR43721*	*piwi-* KD1	0.1
3	chr2L	*hobo*	intron	*beat-IIIc*	*piwi-* KD1	0.1
4	chr2R	*mariner2*	intron	*CG1707*	*piwi-* KD1	0.1
5	chr2R	*invader6*	intron	*Cpr49Ac*	*piwi-* KD1	0.1
6	chr3L	*jockey*	intron	*Dscam4*	*piwi-* KD1	0.2
7	chr3L	*Cr1a*	exon	*Neu2*	*piwi-* KD1	0.2
8	chr3L	*roo*	intron	*CG32447*	*piwi-* KD1	0.1
9	chr3L	*TART-A*	intron	*CG14459*	*piwi-* KD1	0.3
10	chr3L	*Fw3*	intron	*CG41343*	*piwi-* KD1	0.1
11	chr3R	*roo*	intron	*hth*	*piwi-* KD1	0.1
12	chr3R	*roo*	intron	*CG4733*	*piwi-* KD1	0.1
13	chr3R	*412*	intron	*sba*	*piwi-* KD1	0.1
14	chr3R	*jockey*	intergenic	*slo, Ppox*	*piwi-* KD1	0.5
15	chr3R	*Stalker4*	intergenic	*aqrs*	*piwi-* KD1	0.6
16	chr4	*412*	intron	*Actbeta*	*piwi-* KD1	0.2
17	chrX	*springer*	intron	*ptr*	*piwi-* KD1	0.1
18	chrX	*hopper*	intron	*CR44999*	*piwi-* KD1	0.2
19	chrX	*hobo*	intron	*CG42541*	*piwi-* KD1	0.5
20	chrX	*S-element*	intron	*CR45668*	*piwi-* KD1	0.2
21	chrX	*412*	exon	*CG5921*	*piwi-* KD1	0.1
22	chrX	*hobo*	intron	*Pde9*	*piwi-* KD1	0.3
23	chrX	*I-element*	3` UTR	*Neto*	*piwi-* KD1	0.1
24	chrX	*HMS-Beagle*	intron	*acj6*	*piwi-* KD1	0.3
25	chrX	*INE-1*	intron	*shi*	*piwi-* KD1	0.2
26	chrX	*1360*	3` UTR	*ari-1*	*piwi-* KD1	0.1
27	chr2R	*1360*	intron	*Nipped-B*	*piwi-* KD1	0.4
28	chrX	*FB*	intron	*Rip11*	*piwi-* KD1	0.1
29	chr2L	*transib4*	exon	*CG7371*	*piwi* -KD2	0.2
30	chr2L	*ZAM*	5` UTR	*ade3*	*piwi* -KD2	0.2
31	chr2L	*ZAM*	intron	*Pvf3*	*piwi* -KD2	0.1
32	chr2L	*transib3*	intron	*CG40006*	*piwi* -KD2	1.2
33	chr2R	*F-element*	exon	*CG40378*	*piwi* -KD2	1.5
34	chr2R	*Idefix*	intron	*IntS3*	*piwi* -KD2	0.3
35	chr2R	*roo*	intron	*ap*	*piwi* -KD2	0.2
36	chr2R	*ZAM*	intron,3` UTR	*magu, Def*	*piwi* -KD2	0.3
37	chr2R	*Fw2*	exon	*Roc2*	*piwi* -KD2	0.3
38	chr2R	*hopper2*	intron	*fas*	*piwi* -KD2	0.5
39	chr2R	*roo*	intron	*igl*	*piwi* -KD2	0.1
40	chr2R	*BS4*	intron, intron	*HmgZ , HmgD*	*piwi* -KD2	0.4
41	chr3L	*1731*	intron	*CG6175*	*piwi* -KD2	0.1
42	chr3L	*springer*	intron	*rols*	*piwi* -KD2	0.1
43	chr3L	*INE-1*	intron	*barc*	*piwi* -KD2	0.1

44	chr3L	*hobo*	intron	*Ten-m*	*piwi* -KD2	0.1
45	chr3L	*F-element*	intron	*nAChRalpha4*	*piwi* -KD2	0.5
46	chr3R	*Quasimodo*	intron	*CG45076*	*piwi* -KD2	0.2
47	chr3R	*17.6*	intron	*Men*	*piwi* -KD2	0.2
48	chr3R	*jockey*	intron	*Ace*	*piwi* -KD2	0.4
49	chr4	*1731*	intron	*PMCA*	*piwi* -KD2	0.1
50	chrX	*roo*	intron	*CG3655*	*piwi* -KD2	0.1
51	chrX	*roo*	intron	*CG32694*	*piwi* -KD2	0.1
52	chrX	*412*	intron	*RhoU*	*piwi* -KD2	0.1
53	chrX	*Juan*	intron	*Tis11*	*piwi* -KD2	0.3
54	chr2L	*hobo*	intron	*CG31808*	*piwi* -KD1 & -KD2	0.1 & 0.1
55	chr2L	*roo*	3` UTR	*tj*	*piwi* -KD1 & -KD2	0.1 & 0.1
56	chr2L	*Juan*	intron	*sick*	*piwi* -KD1 & -KD2	0.2 & 0.3
57	chr2L	*Quasimodo*	intron	*CR44909*	*piwi* -KD1 & -KD2	0.1 & 0.2
58	chr2R	*springer*	intron	*ap*	*piwi* -KD1 & -KD2	0.3 & 0.3
59	chr2R	*hobo*	3` UTR	*CR44276*	*piwi* -KD1 & -KD2	0.1 & 0.1
60	chr2R	*Dm88*	intron	*kn*	*piwi* -KD1 & -KD2	0.1 & 0.1
61	chr3L	*Tc3*	intron	*CG3967*	*piwi* -KD1 & -KD2	0.2 & 0.2
62	chr3L	*hobo*	intergenic	*CR44672*	*piwi* -KD1 & -KD2	0.1 & 0.1
63	chr3R	*Transpac*	exon	*Irc*	*piwi* -KD1 & -KD2	0.2 & 0.2
64	chrX	*297*	intron	*ct*	*piwi* -KD1 & -KD2	0.2 & 0.1
65	chrX	*roo*	intron	*CG33181*	*piwi* -KD1 & -KD2	0.3 & 0.4
66	chrX	*flea*	intron	*CG32683*	*piwi* -KD1 & -KD2	0.4 & 0.2
67	chrX	*F-element*	intron	*spri*	*piwi* -KD1 & -KD2	0.1 & 0.2
68	chrX	*opus*	intron	*CG43155*	*piwi* -KD1 & -KD2	0.2 & 0.2
69	chrX	*roo*	intron	*mnb*	*piwi* -KD1 & -KD2	0.3 & 0.2
70	chrX	*roo*	intron	*Hers*	*piwi* -KD1 & -KD2	0.3 & 0.3

New transposon insertions in the genome of *piwi*-KD niche cells (CpCs and TFs) of 2-week-old *bab1>gfp;piwi*$^{RNAi-1}$ flies were indentified by comparing with the genomic sequences of 2-week-old GFP-negative ovarian non-niche somatic cells and of one-day-old ovary of *bab1>gfp;piwi*$^{RNAi-1}$ flies . knockdown (KD)1 and KD2 represent two biological replicates of genomic seqencing from *piwi*-KD niche cells.

Column C-TE (transposable element) shows TE types. Red, retrotransposon; blue, DNA transposon.

Column D-Insertion classification shows the retrotransposon insertion site either in the exon, intron, 5'-untranslated region (UTR), 3'-UTR or the intergenic region.

Column E-Gene name shows genes inserted by retrotransposons. Note that the envent No. 36 and 40 affects two genes that are using the same seqences but with different orrientation.

Column F-Attribute indicates the event observed in *piwi*-KD1 CpCs, *piwi*-KD2 CpCs, or both.

Column G-Coverage ratio (CR) represents the relative occurrence rate of jumping events by caculation of TE insertion reads over the number of control genome mapped reads plus a one pseudocount read. For instance, 2 TE insertion reads and 4 reads from contrl genome, the CR will be (2/4+1) =0.4, high CR number means high jumpning rate.

Supplementary table 3. Expression of retrotransposons in piwi-KD cap cells

	TE symbol	Average FPKM (N=2) in cap cells *bab1>gfp*	Average FPKM (N=2) in cap cells *bab1>gfp, piwi* $^{RNAi-1}$	Fold change
1	**ZAM**	80.4107	3760.035	46.760
2	**Stalker2**	295.878	4101.565	13.862
3	**HeT-A**	50.76195	676.7065	13.331
4	**mdg1**	2574.28	27375.3	10.634
5	**blood**	462.0415	4108.64	8.892
6	**I-element**	157.7935	968.4895	6.138
7	**NOF**	3.04711	16.9873	5.575
8	**297**	3749.53	19831.4	5.289
9	**412**	444.3565	2137.225	4.810
10	**TAHRE**	30.3846	133.4706	4.393
11	**gypsy5**	122.68365	525.8695	4.286
12	**Osvaldo**	0.992615	4.16495	4.196
13	**micropia**	188.316	772.128	4.100
14	**Quasimodo**	473.0165	1790.857	3.786
15	**flea**	1564.22	5861.84	3.747
16	**gypsy8**	2.393175	8.9083	3.722
17	**pogo**	621.7705	1693.4	2.724
18	**transib2**	27.52165	70.61905	2.566
19	**P-element**	278.9595	553.5985	1.985
20	**G4**	19.4104	38.12705	1.964
21	**S2**	1.650435	3.233255	1.959
22	**Stalker**	150.632	277.8145	1.844
23	**Tirant.**	189.524	329.69	1.740
24	**Stalker4**	338.634	581.6805	1.718
25	**HB**	34.15335	54.6412	1.600
26	**transib1**	22.04081	33.472	1.519
27	**diver**	1001.8635	1463.278	1.461
28	**1731**	3164.015	4263.205	1.347
29	**looper1**	8.30462	11.02255	1.327
30	**Dyak\TART**	246.294	326.8525	1.327
31	**S-element**	40.98625	53.5653	1.307
32	**Tabor.**	2154.81	2814.6	1.306
33	**Doc3-element**	380.6885	496.2225	1.303
34	**jockey**	1037.623	1347.285	1.298
35	**gypsy**	2926.44	3711.695	1.268
36	**hobo**	1502.71	1903.785	1.267
37	**G7**	12.129415	15.22255	1.255
38	**17.6**	6694.725	8375.83	1.251
39	opus	691.79	863.4185	1.248
40	INE-1	19.3847	24.1005	1.243
41	gtwin	81.627	97.5031	1.194
42	Tom1	212.111	252.389	1.190
43	Dana\Tom	0.58502	0.694375	1.187
44	HMS-Beagle2	287.0065	339.62	1.183
45	G6	641.4905	752.263	1.173
46	Fw2	15.331555	17.316065	1.129
47	copia	45375.75	49536.55	1.092
48	diver2	35.5142	37.97105	1.069
49	mdg3	2059.15	2198.54	1.068
50	mariner2	51.7793	54.20355	1.047

51	1360	481.7515	488.2905	1.014
52	roo	3113.22	3153.885	1.013
53	rooA	385.6845	388.7205	1.008
54	BS	207.838	207.517	0.998
55	gypsy6	120.577	119.391	0.990
56	Burdock	313.794	304.973	0.972
57	Juan	318.0455	308.2885	0.969
58	jockey2	24.6618	23.7219	0.962
59	Porto1	38.1967	36.19655	0.948
60	invader6	9367.565	8749.985	0.934
61	gypsy10	5.938955	5.476325	0.922
62	G2	951.575	874.2475	0.919
63	Tc1	101.93115	92.9939	0.912
64	Tc1-2	51.705	46.9418	0.908
65	Doc	9568.515	8662.32	0.905
66	G3	2.772655	2.4989015	0.901
67	HMS-Beagle	180.6835	159.7965	0.884
68	gypsy2	326.402	287.504	0.881
69	Idefix	885.6925	770.8025	0.870
70	FB	599.6765	517.9275	0.864
71	invader1	1160.986	1001.28	0.862
72	invader2	898.9295	756.8805	0.842
73	F-element	4031.785	3326.065	0.825
74	Transpac	745.166	614.7145	0.825
75	Cr1a	477.069	386.9535	0.811
76	G5A	16.9442	13.68275	0.808
77	Ivk	631.872	496.171	0.785
78	Dm88	1220.2685	948.7215	0.777
79	Rt1c	0.4638445	0.3547345	0.765
80	Dsim\ninja	69.6569	52.9757	0.761
81	Bari2	33.9348	25.61635	0.755
82	gypsy4	838.5355	629.3965	0.751
83	gypsy11	17.67805	13.16625	0.745
84	aurora-element	72.2216	53.70605	0.744
85	G5	60.52425	44.68655	0.738
86	invader3	1636.12	1199.245	0.733
87	3S18	14306.15	10468.215	0.732
88	invader4	3455.615	2469.605	0.715
89	Tc3	49.7769	35.55685	0.714
90	springer	4862.205	3400.255	0.699
91	Doc2-element	368.3405	255.9865	0.695
92	gypsy12	14.68287	10.15084	0.691
93	baggins	169.076	115.266	0.682
94	hopper2	83.9809	57.01335	0.679
95	G-element	78.1874	51.6365	0.660
96	Stalker3	1345.2915	888.321	0.660
97	Max-element	495.2785	326.475	0.659
98	transib3	59.96495	38.18615	0.637
99	Rt1a	149.577	93.32425	0.624
100	Q-element	200.083	119.1245	0.595
101	Circe	178.0035	102.7874	0.577
102	Helena	16.27045	9.38524	0.577
103	McClintock	74.8748	42.41885	0.567
104	Rt1b	732.008	391.8535	0.535

105	frogger	41.156	20.5236	0.499
106	GATE	629.86	306.9	0.487
107	hopper	2673.77	1257.43	0.470
108	R1A1-element	1256.395	587.4755	0.468
109	gypsy3	39.3584	17.91985	0.455
110	rover	1138.5375	447.7865	0.393
111	gypsy9	1.027961	0.352931	0.343
112	accord2	24.97515	8.40448	0.337
113	R2-element	8694.495	2911.61	0.335
114	Bari1	224.372	73.2829	0.327
115	transib4	6.72327	1.81243	0.270
116	X-element	2189.535	562.192	0.257
117	Fw3	1.28323	0.316629	0.247
118	gypsy7	16.3924	3.36859	0.205
119	BS3	6.74534	1.086035	0.161
120	accord	926.7545	116.18565	0.125
121	invader5	18.55825	0.4892035	0.026

Supplementary table 4. Toll-like receptor (TLR) ortholog in *Drosophila*

Human Symbol	PAMPs	Fly Symbol	DIOPT Ranking	DIOPT Score	Best Score	Best Score Reverse	Prediction Derived From
TLR3	Viral dsRNA	*Toll-7*	high	4	Yes	Yes	eggNOG, OrthoDB, Panther, TreeFam
		Tollo	high	4	Yes	Yes	eggNOG, Homologene, OrthoDB, TreeFam
TLR4	Viral Envelope glycoproteins, LPS	*Tehao (Toll-5)*	high	4	Yes	Yes	eggNOG, OrthoDB, orthoMCL, Panther
TLR7	Viral ssRNA	*Tl (Toll)*	high	3	Yes	Yes	eggNOG, OrthoDB, TreeFam
		18w	high	3	Yes	Yes	eggNOG, OrthoDB, TreeFam
TLR9	Viral CpG DNA	*Tl (Toll)*	high	3	Yes	Yes	eggNOG, OrthoDB, TreeFam
		18w	high	3	Yes	Yes	eggNOG, OrthoDB, TreeFam
TLR10	Triacylated lipopeptides	*Toll-9*	high	8	Yes	Yes	Inparanoid, OrthoDB, OrthoFinder, OrthoInspector, Panther, Phylome, RoundUp, TreeFam

Note that this table showed all hign-ranking ortholog of TLRs in *Drosophila melangaster*

Data from DIPOT (https://www.flyrnai.org/cgi-bin/DRSC_orthologs.p

PAMPs : Pathogen-Associated Molecular Patterns

LPS : Lipopolysaccharides
