# Supplementary material for: Piwi reduction in the aged niche eliminates germline stem cells via Toll-GSK3 signaling
Source: Nat Commun. 2020 Jun 19;11:3147. doi: 10.1038/s41467-020-16858-6 (PMC7305233; doi:10.1038/s41467-020-16858-6)
Supplement: Supplementary file 3 — Reporting Summary [file 41467_2020_16858_MOESM3_ESM.pdf]

nature research

Corresponding author(s): Hwei-Jan Hsu

Last updated by author(s): May 16, 2020

Reporting Summary

Nature Research wishes to improve the reproducibility of the work that we publish. This form provides structure for consistency and transparency in reporting. For further information on Nature Research policies, see Authors & Referees and the Editorial Policy Checklist .

Statistics

For all statistical analyses, confirm that the following items are present in the figure legend, table legend, main text, or Methods section.

n/a	Confirmed	
☐	☒	The exact sample size (*n*) for each experimental group/condition, given as a discrete number and unit of measurement
☐	☒	A statement on whether measurements were taken from distinct samples or whether the same sample was measured repeatedly
☐	☒	The statistical test(s) used AND whether they are one- or two-sided<br>*Only common tests should be described solely by name; describe more complex techniques in the Methods section.*
☒	☐	A description of all covariates tested
☐	☒	A description of any assumptions or corrections, such as tests of normality and adjustment for multiple comparisons
☐	☒	A full description of the statistical parameters including central tendency (e.g. means) or other basic estimates (e.g. regression coefficient) AND variation (e.g. standard deviation) or associated estimates of uncertainty (e.g. confidence intervals)
☐	☒	For null hypothesis testing, the test statistic (e.g. *F*, *t*, *r*) with confidence intervals, effect sizes, degrees of freedom and *P* value noted<br>*Give P values as exact values whenever suitable.*
☒	☐	For Bayesian analysis, information on the choice of priors and Markov chain Monte Carlo settings
☒	☐	For hierarchical and complex designs, identification of the appropriate level for tests and full reporting of outcomes
☒	☐	Estimates of effect sizes (e.g. Cohen's *d*, Pearson's *r*), indicating how they were calculated

Our web collection on statistics for biologists contains articles on many of the points above.

Software and code

Policy information about availability of computer code

Data collection	Confocal Images were acquired using Zen softwares (2010 version) on Zeiss LSM710 confocal microscope.
Data analysis	We used Image J (version 1.50i) for image analysis (doi:10.1038/nmeth.2019), LSM image browser (version 4,2,0,121) for modification of confocal images (https:// www.embl.de/eamnet/html/body_image_browser.html), Microsoft-Excel software for two-tailed student t-test and chi-square test of statistics and making graph. GraphPad Prism 8 software (version 8.3.0)(MachineID : B370B245D17) were used for making graph and One-way ANOVA. 3D reconstructions were made from confocal z-sections using Imaris (version x64, 9.1.2)(Bitplane)

For manuscripts utilizing custom algorithms or software that are central to the research but not yet described in published literature, software must be made available to editors/reviewers. We strongly encourage code deposition in a community repository (e.g. GitHub). See the Nature Research guidelines for submitting code & software for further information.

Data

Policy information about availability of data

All manuscripts must include a data availability statement. This statement should provide the following information, where applicable:

- Accession codes, unique identifiers, or web links for publicly available datasets
- A list of figures that have associated raw data
- A description of any restrictions on data availability

Transposon expression profiles (Raw FPKM) done by RNA-seq from sorted niche cell are provided in Supplementary Table 3 of Supplementary Information section. All other data supporting the findings of this study are available from the responding author upon reasonable request.

Field-specific reporting

Please select the one below that is the best fit for your research. If you are not sure, read the appropriate sections before making your selection.

☒ Life sciences ☐ Behavioural & social sciences ☐ Ecological, evolutionary & environmental sciences

For a reference copy of the document with all sections, see nature.com/documents/nr-reporting-summary-flat.pdf

Life sciences study design

All studies must disclose on these points even when the disclosure is negative.

Sample size	Sample size, number of replicates, error bars and statistical tests were chosen based on accepted practices in the field and stated in each figur e legend. Generally, experiments were performed independently and reproduced using at least two biological replicates.
Data exclusions	No data were excluded from the analysis.
Replication	At least two biological replicates were perform. Exact numbers are mentioned in respective figures or figure legends
Randomization	This is not relevant for this study since it relies on the description of a cellular dynamics coupled with dissections experiments which can not be randomized. Control samples were always treated in the exact same condition as the experimental group (stage, culture, time frame of observation, time of sample illumination).
Blinding	This is not relevant for this study since it relies on the description of a cellular dynamics coupled with dissections experiments which can not be blinded.

Reporting for specific materials, systems and methods

We require information from authors about some types of materials, experimental systems and methods used in many studies. Here, indicate whether each material, system or method listed is relevant to your study. If you are not sure if a list item applies to your research, read the appropriate section before selecting a response.

Materials & experimental systems

n/a	Involved in the study	
☐	☒	Antibodies
☐	☒	Eukaryotic cell lines
☒	☐	Palaeontology
☐	☒	Animals and other organisms
☒	☐	Human research participants
☒	☐	Clinical data

Methods

n/a	Involved in the study	
☒	☐	ChIP-seq
☐	☒	Flow cytometry
☒	☐	MRI-based neuroimaging

Antibodies

Antibodies used	Primary antibodies obtained from Developmental Studies Hybridoma Bank (DSHB) were: mouse anti-Hts, mouse anti-Lamin (Lam) C (1:25), rat anti-E-Cad (DCAD2, 1:3), , mouse anti-Cactus antibody (1:500; 3H12), mouse anti-Dorsal antibody (1:100; 7A4) and mouse anti-Arm (N27A1, 1:4). Rabbit anti-pMad antibody (1:200; #1880,<br>Epitomics), mouse anti-beta-gal antibody (1:500; Promega), mouse anti-Piwi antibody (1:1000; non-commercialized antibody, a gift from Dr. Mikiko C. Siomi, University of Tokyo, Japan), guinea pig anti-Traffic jam (Tj) antibody (1:5000; a gift from Dr. Dorothea Godt, University of Toronto, Canada), rabbit anti-Histone H2AvD pS137antibody (1:1000; #600-401-914, Rockland), mouse anti-GSK3alpha/beta antibody(1:200; MA3-038, Thermo Fisher Scientific), rabbit anti-pSer9-GSK3-beta antibody(1:200; #5558, Cell Signaling), mouse anti-pTyr216-GSK3 antibody(1:150, #05-413, Millipore), and rabbit anti-GFP antibody(1:1000; Torrey Pines). Mouse anti-ENV antibody (1:100, a gift from Dr. Joshua Dubnau, Stony Brook University School of Medicine, U.S.A.<br><br>For Secondary antibodies, AlexaFluor 488, 568 or 633-conjugated goat anti-mouse, anti-rabbit or anti-rat secondary antibodies (1:500; Molecular Probes) were used for appropriate primary antibodies.
Validation	Primaries antibodies were previously described:<br>1. Mouse anti-Hts, mouse anti-Lamin (Lam) C, rat anti-E-Cad (DCAD2)(N27A1), rabbit anti-pMad, mouse anti-beta-gal and rabbit anti-GFP antibodies: Tseng et al, 2014(PLoS Genet.) doi: 10.1371/journal.pgen.1004888.<br>2. Mouse anti-Arm : In Supplementary figure 7a of this manuscript.<br>3. Mouse anti-Piwi antibody : Saito et al, 2009(Nature)doi: 10.1038/nature08501.<br>4. Guinea piganti-Traffic jam (Tj) antibody : Lai et al, 2017(J Cell Biol.) doi: 10.1083/jcb.201610063<br>5. Rabbit anti-Histone H2AvD pS137 antibody : Kao et al., 2014(Aging Cell) doi: 10.1111/acel.12288.

6. Mouse anti-GSK3alpha/beta antibody : MA3-038, Thermo Fisher Scientific, https://www.thermofisher.com/order/genome-database/dataSheetPdf?producttype=antibody&productsubtype=antibody_primary&productId=MA3-038
7. rabbit anti-pSer9-GSK3-beta antibody(Cell Signaling): Gärtner et al., 2006(J Cell Sci.) doi: 10.1242/jcs.03159
8. Mouse anti-pTyr216-GSK3 antibody(Millipore) : #05-413, Millipore, file:///C:/Users/wawa/Downloads/05-413-15648.pdf
9. mouse anti-Cactus antibody (1:500; 3H12), mouse anti-Dorsal antibody (1:100; 7A4) :M. Fontenele et al., 2013(Mol Biol Cell)
10. Mouse anti-ENV antibody : Chang et al. 2019 (Curr Biol.)

Eukaryotic cell lines

Policy information about cell lines

Cell line source(s)	Human embryonic kidney (HEK) 293-derived CG cells (Yung-Feng Liao's laboratory, one of our co-author). Reference : Liao et al., 2004(J Biol Chem.)
Authentication	HEK 293-derived CG cells were tested for the ability on production of Abeta40 peptides in Supplementary figure 17b.
Mycoplasma contamination	HEK 293-derived CG cells were not tested for mycoplasma contamination when we cultured this cell line in our laboratory because of no signs regarding mycoplasma contamination; however, we can not rule out this possibility before we perform the test for mycoplasma contamination.
Commonly misidentified lines (See ICLAC register)	No cell lines used are listed in the database of commonly misidentified cell lines.

Animals and other organisms

Policy information about studies involving animals; ARRIVE guidelines recommended for reporting animal research

Laboratory animals	Drosophila melanogaster were used in this study.
Wild animals	The study did not involve wild animals
Field-collected samples	The study did not involve the samples collected from the field.
Ethics oversight	No ethical approval is needed to do research on Drosophila melanogaster.

Note that full information on the approval of the study protocol must also be provided in the manuscript.

Flow Cytometry

Plots

Confirm that:

- [x] The axis labels state the marker and fluorochrome used (e.g. CD4-FITC).
- [x] The axis scales are clearly visible. Include numbers along axes only for bottom left plot of group (a 'group' is an analysis of identical markers).
- [x] All plots are contour plots with outliers or pseudocolor plots.
- [x] A numerical value for number of cells or percentage (with statistics) is provided.

Methodology

Sample preparation	Ovarian cells of Drosophila were dissociated enzymatically to separate different cell types in ovary tissue for niche cell sorting. Detail sample preparation listed in Methods
Instrument	FACSAriaII (BD Biosciences).
Software	BD FACSDiva software v6.2 for analysis
Cell population abundance	Around 20,000 (0.1-0.2% of total cells) GFP-positive niche cells were sorted per experiment (100 ovaries). Purity of sorted GFP-positive niche cells was identified by immunostaining for GFP (sorting marker), Tj (soma marker) and LamC (niche marker) using confocal microscope.
Gating strategy	GFP positivity threshold (using GFP-H and GFP-W) was defined on the basis of the control sample which does not carry GFP-positive cells. Identical positivity threshold was applied to all samples of fruit flies carrying GFP-positive niche cells.

- [x] Tick this box to confirm that a figure exemplifying the gating strategy is provided in the Supplementary Information.
